# Socioeconomic differences in healthcare expenditure and utilization in The Netherlands

**DOI:** 10.1186/s12913-021-06694-9

**Published:** 2021-07-03

**Authors:** Bette Loef, Iris Meulman, Gerrie-Cor M. Herber, Geert Jan Kommer, Marc A. Koopmanschap, Anton E. Kunst, Johan J. Polder, Albert Wong, Ellen Uiters

**Affiliations:** 1 National Institute for Public Health and the Environment, Bilthoven, The Netherlands; 2 Erasmus School of Health Policy and Management, Erasmus University Rotterdam, Rotterdam, The Netherlands; 3 Amsterdam UMC, Department of Public and Occupational Health, University of Amsterdam, Amsterdam Public Health research institute, Amsterdam, The Netherlands; 4 Tilburg School of Social and Behavioral Sciences, Tranzo, Tilburg University, Tilburg, The Netherlands

**Keywords:** Education, Health, Healthcare expenditure, Healthcare utilization, Income, Socioeconomic status

## Abstract

**Background:**

Worldwide, socioeconomic differences in health and use of healthcare resources have been reported, even in countries providing universal healthcare coverage. However, it is unclear how large these socioeconomic differences are for different types of care and to what extent health status plays a role. Therefore, our aim was to examine to what extent healthcare expenditure and utilization differ according to educational level and income, and whether these differences can be explained by health inequalities.

**Methods:**

Data from 18,936 participants aged 25–79 years of the Dutch Health Interview Survey were linked at the individual level to nationwide claims data that included healthcare expenditure covered in 2017. For healthcare utilization, participants reported use of different types of healthcare in the past 12 months. The association of education/income with healthcare expenditure/utilization was studied separately for different types of healthcare such as GP and hospital care. Subsequently, analyses were adjusted for general health, physical limitations, and mental health.

**Results:**

For most types of healthcare, participants with lower educational and income levels had higher healthcare expenditure and used more healthcare compared to participants with the highest educational and income levels. Total healthcare expenditure was approximately between 50 and 150 % higher (depending on age group) among people in the lowest educational and income levels. These differences generally disappeared or decreased after including health covariates in the analyses. After adjustment for health, socioeconomic differences in total healthcare expenditure were reduced by 74–91 %.

**Conclusions:**

In this study among Dutch adults, lower socioeconomic status was associated with increased healthcare expenditure and utilization. These socioeconomic differences largely disappeared after taking into account health status, which implies that, within the universal Dutch healthcare system, resources are being spent where they are most needed. Improving health among lower socioeconomic groups may contribute to decreasing health inequalities and healthcare spending.

**Supplementary Information:**

The online version contains supplementary material available at 10.1186/s12913-021-06694-9.

## Background

Good health and universal access to high-quality healthcare are important drivers of human welfare and well-functioning societies [1, 2]. However, worldwide, there are large and persistent differences in health by socioeconomic status [3–5]. Education and income are important indicators of socioeconomic status that are frequently applied in public health research [6]. People with lower educational and income levels have recurrently been found to have a higher risk of developing health problems and an increased mortality rate [3–5]. Even in high-income countries with universal coverage systems, large health gaps in (healthy) life expectancy exist by socioeconomic status [4]. Furthermore, although efforts have been made to reduce these health inequalities, studies examining trends in socioeconomic health differences in Europe indicate that they are rather persistent [4, 7, 8].

As poorer health is related to higher healthcare expenditures [9], socioeconomic inequalities in health may contribute to a skewed distribution of the use of healthcare resources between socioeconomic groups. Correspondingly, multiple studies from different high-income countries with different healthcare systems have reported that people with low socioeconomic status have disproportionally higher healthcare expenditure and utilization [10–14]. A lower educational and income level has been associated with paying more visits to the General Practitioner (GP), having more hospital admissions, and using more prescribed medication [14–16]. Additionally, multiple studies found that inequalities in health status explain (part of the) socioeconomic differences in healthcare expenditure and utilization [14–18]. After adjustment for health, lower socioeconomic groups even make less use of some types of healthcare [15–18]. For example, hospital care and dental care have been shown to be used less often by lower socioeconomic groups after taking into account health status [16–18]. These findings have consistently been found across different countries, including those with universal coverage systems, even though the characteristics of the healthcare system (i.e. characteristics concerning health financing, healthcare delivery, and governance and resource allocation [19]) also influence the existence of socioeconomic differences in the use of healthcare services in itself [20–22]. In the Dutch healthcare system, all residents are entitled to a comprehensive basic health insurance package under the Health Insurance Act [23]. When consuming healthcare (except for GP care and maternity care), a deductible applies, as well as possible co-payments. The Dutch government provides healthcare benefits to contribute towards the costs of the healthcare insurance of people with a low income. In addition to the mandatory basic package, a supplementary voluntary insurance package covering healthcare such as dental care and physiotherapeutic care can be purchased. However, co-payments may still apply to care covered in the supplementary insurance package. Long-term stay in for example a nursing home is covered under different legislation [23]. It should be noted that virtually all health insurance companies in the Netherlands are not-for-profit cooperatives that allocate any profits they make to the reserves they are required to maintain or return them in the form of lower premiums [24].

Despite the available literature that gives some insight into general patterns, the extent of socioeconomic differences in healthcare expenditure and utilization for specific types of healthcare in countries with universal healthcare coverage like the Netherlands is currently unknown. In the Netherlands, an extensive overview into socioeconomic differences in healthcare utilization was published in 2007 using data from 2003 [16]. However, details on the current socioeconomic differences in healthcare utilization, and resulting healthcare expenditure, are lacking. Therefore, this study aims to examine to what extent healthcare expenditure and utilization for several types of care differ according to educational level and income in the Netherlands, and whether these differences can be explained by a poorer health status among lower socioeconomic groups.

## Methods

### Study design and population

In this cross-sectional study, data were used from the Dutch Health Interview Survey (DHIS) 2016, 2017, and 2018 [25]. The DHIS is conducted annually by Statistics Netherlands among a random sample of persons aged 0 years and older living in private households (n ≈ 9,500), who are selected from the Personal Records Database. Response rates in 2016, 2017, and 2018 were approximately 60 %. In this survey, information on health, medical contacts, lifestyle, and preventive behavior is collected. In the current study, persons participating in DHIS in 2016, 2017, or 2018 were included to have sufficient power across all socioeconomic groups. The DHIS 2016–2018 data were linked at the individual level to data on age, sex, educational level, and household income from Statistics Netherlands, and to data on healthcare expenditure obtained from Vektis [26]. Age and sex were based on Dutch population registration (BRP) data, educational level was based on registry data from educational institutes and the Labor Force Survey, and household income was based on tax registration data. The healthcare expenditure data were derived from registered claims data that included all healthcare expenditure covered by the basic insurance package in 2017. Statistics Netherlands functioned as a trusted third party, enabling the linkage between the datasets, while ensuring the privacy of the involved participants, according to Dutch law (Statistics Netherlands Act 2003). For the current study, the population aged 25–79 years in DHIS 2016–2018 was selected. Since the current study was aimed at the general adult population and the used socioeconomic status indicators may be less applicable to children/adolescents and elderly adults, age groups below 25 years and above 79 years were excluded.

### Measures

#### Healthcare expenditure

As an outcome measure, total healthcare expenditure in 2017 was used. This includes the total sum of registered healthcare expenditure in Euros covered by the comprehensive basic insurance package. Furthermore, the following types of healthcare expenditure were taken into account separately: GP care (excluding registration fees), hospital care, dental care, mental health care, physiotherapeutic care, home care, pharmaceutical care, and maternity home care.

#### Healthcare utilization

In DHIS, participants were asked whether they consulted a GP, medical specialist, dentist, mental health care professional (psychologist, psychiatrist, psychotherapist), and physiotherapist, and whether they stayed in the hospital (either overnight or during a day admission) and received home care in the last 12 months. Subsequently, they were asked about the number of GP, medical specialist, and dentist consultations in the past 4 weeks, and the number of overnight hospital admissions, daytime hospital admissions, and the number of consultations with a mental health care professional and physiotherapist in the last 12 months. In the Netherlands, a medical specialist can only be consulted after referral from a GP who acts as a gatekeeper.

#### Educational level and income

Educational level was operationalized as the highest obtained educational degree. Participants were divided into four categories: low (primary education or less), low-moderate (lower vocational education or lower secondary education), moderate-high (intermediate vocational education or higher secondary education), and high (higher vocational education or university) educational level. Income was operationalized as disposable household income, standardized for the size and composition of the household [27]. Income was divided into quintiles, with the lowest quintile representing the lowest incomes, and the highest quintile representing the highest incomes.

### Covariates

Age (in 5-year age groups), sex (male, female), and year in which the DHIS was completed (2016, 2017, 2018) were included as covariates. To adjust for health status, three variables were used: perceived general health (on a 5-point scale from very good to very poor), number of physical limitations (based on the Organization for Economic Co-operation and Development (OECD) indicator consisting of 7 items about to what extent the participant experiences limitations with hearing, sight, and motor functioning [28]), and mental health (healthy vs. unhealthy, based on the 5 items from the Mental Health Inventory 5 using a cutoff of 60 points [29]). The measure of perceived general health has been found to be a robust predictor of healthcare utilization and mortality, even after adjustment for clinical parameters [17, 30].

### Statistical analysis

Because the association between socioeconomic status and healthcare expenditure/utilization is likely to differ by age [5], analyses were conducted separately for the following age groups: 25–44 years, 45–64 years, and 65–79 years. Descriptive information on age, sex, educational level, income, health, healthcare expenditure, and healthcare utilization were presented for these three groups.

All healthcare expenditure and utilization variables, except for total healthcare and GP care expenditure, generally followed a mixed distribution, i.e. a continuous distribution with the bulk of the observations at zero [31]. Because a substantial proportion of the participants did not have healthcare expenses for specific types of healthcare or did not use specific types of healthcare, this resulted in an excess of zeros. Furthermore, the healthcare expenditure and utilization variables among the participants with healthcare expenditure/utilization followed a positively skewed distribution, because most participants used limited healthcare resources and a small proportion of the participants used a high amount of healthcare resources. Therefore, two-part models were used to study socioeconomic differences in healthcare expenditure and utilization. In the first part, logistic regression analysis was used to estimate differences in the probability of having healthcare expenditure or using healthcare (yes vs. no), expressed as odds ratios (ORs). In the second part, generalized linear models (GLM) were used to estimate differences in the amount of healthcare expenditure/utilization among the participants who used healthcare resources [31], expressed as rate ratios (RRs). For the healthcare expenditure variables (in Euros), GLM with log link function and a gamma distribution was applied [32]. Because the healthcare utilization variables were count variables operationalized as the number of consultations or admissions, GLM with log link function and a negative binomial distribution was used for these variables. To support the interpretation of the ORs and RRs, effect estimates in the original scale of the outcome variables adjusted for covariates were computed using estimated marginal means.

For every outcome measure, two analyses were conducted for educational level and income separately. The first analysis was adjusted for potential confounders age, sex, the interaction between age and sex, and the year in which the DHIS was completed (i.e. 2016–2018). The second analysis was additionally adjusted for perceived general health, physical limitations, and mental health to examine whether these potential mediators could explain possible socioeconomic differences in healthcare expenditure and utilization.

Analyses were conducted using IBM SPSS Statistics, V.25.0 (IBM Corp, New York).

## Results

### Study population

**Fig. 1:**
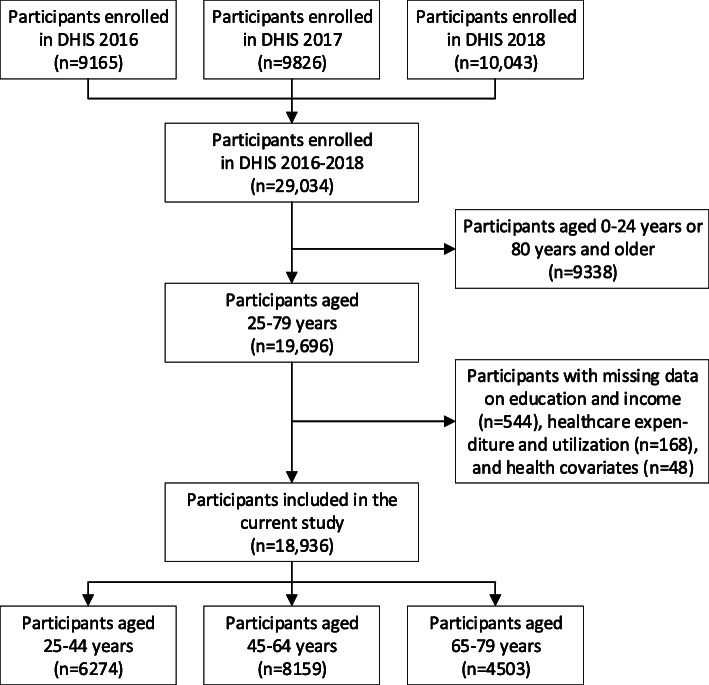
Flowchart of study participants. DHIS, Dutch Health Interview Survey

In total, 29,034 participants enrolled in DHIS 2016–2018. For the current study, participants aged 25–79 years with complete data on educational level, income, healthcare expenditure, healthcare utilization, and health covariates were included. This resulted in a study population of 6274 participants aged 25–44 years (mean age 34.8 years (SD 5.8)), 8159 participants 45–64 years (mean age 54.6 years (SD 5.7)), and 4503 participants aged 65–79 years (mean age 70.8 years (SD 4.1)) (Fig. 1). Table 1 shows the characteristics of the study population. Approximately half of the population (51.0 %) was female. The proportion of participants who obtained the highest educational level was on average higher in the younger compared to the older age groups. Among all age groups, most participants (between 67.5 and 84.3 %) rated their general health as (very) good and most participants (between 81.7 and 95.0 %) did not report physical limitations.

**Table 1:** Characteristics of the study population (*n* = 18,936)

	Participants aged 25–44 years (*n* = 6274)		Participants aged 45–64 years (*n* = 8159)		Participants aged 65–79 years (*n* = 4503)		Total study population (*n* = 18,936)	
	*%*	*n*	*%*	*n*	*%*	*n*	*%*	*n*
Age in years, mean and SD	34.8	5.8	54.6	5.7	70.8	4.1	51.9	14.6
Sex (% female)	52.1	3271	50.9	4150	49.6	2235	51.0	9656
Highest obtained educational level (%)								
Low	3.8	237	7.4	600	14.0	630	7.7	1467
Low-moderate	10.2	643	18.7	1524	31.0	1395	18.8	3562
Moderate-high	41.9	2628	42.6	3478	32.3	1455	39.9	7561
High	44.1	2766	31.3	2557	22.7	1023	33.5	6346
Year of health survey (%)								
2016	31.2	1960	30.4	2481	30.3	1364	30.7	5805
2017	31.5	1978	32.4	2647	36.8	1656	33.2	6281
2018	37.2	2336	37.1	3031	32.9	1483	36.2	6850
Perceived general health (%)								
Very good	27.2	1708	19.5	1589	14.2	639	20.8	3936
Good	57.0	3578	53.7	4379	53.3	2399	54.7	10,356
Moderate	12.4	779	20.3	1655	25.9	1165	19.0	3599
Poor	2.9	180	5.6	457	5.9	265	4.8	902
Very poor	0.5	29	1.0	79	0.8	35	0.8	143
Number of physical limitations								
0	95.0	5963	87.7	7155	81.7	3681	88.7	16,799
1	3.3	204	6.5	532	9.1	410	6.1	1146
2 or more	1.7	107	5.8	472	9.1	412	5.2	991
Mental health (% unhealthy)	11.1	696	11.3	923	8.7	394	10.6	2013

### Descriptive information on healthcare expenditure and utilization

Table 2 presents an overview of the descriptive information on healthcare expenditure and utilization among the three age groups. Virtually all participants had healthcare expenditure covered by the basic insurance package in 2017. The 0.3 % (n = 58) of the study population who did not have healthcare expenditure covered in 2017 were predominantly young males (mean age 39.4 years) with a high educational level who were in good health (91 % reported (very) good general health). More than 99 % of the participants had expenditure for GP care. After GP care, expenditure for pharmaceutical care and hospital care was most frequent among participants. Among those with positive expenditure, expenditure was highest for mental health care and home care, and lowest for GP care. For all types of healthcare except mental health care (and maternity care), the percentage of those with costs was highest among the oldest age group.

**Table 2:** Descriptive information on healthcare expenditure and utilization among the study population (*n* = 18,936)

	Participants aged 25–44 years (*n* = 6274)			Participants aged 45–64 years (*n* = 8159)			Participants aged 65–79 years (*n* = 4503)			Total study population (*n* = 18,936)		
	*Mean or %*	*SD or n*	*Median*	*Mean or %*	*SD or n*	*Median*	*Mean or %*	*SD or n*	*Median*	*Mean or %*	*SD or n*	*Median*
**Healthcare expenditure**												
Total healthcare expenditure (% yes)	99.3	6233		99.8	8144		100.0	4501		99.7	18,878	
Expenditure in Euros among those with expenditure	1561	5291	202	2361	7901	443	4358	11,100	1368	2573	8165	501
General practitioner care (% yes)	99.1	6220		99.6	8128		99.6	4485		99.5	18,833	
Expenditure in Euros among those with expenditure	77	91	47	85	100	55	115	127	75	89	106	56
Hospital care (% yes)	51.7	3242		65.2	5321		86.5	3896		65.8	12,459	
Expenditure in Euros among those with expenditure	1518	4992	375	2244	7773	476	3107	9312	734	2325	7730	501
Dental care (% yes)	0.7	42		3.2	260		8.2	367		3.5	669	
Expenditure in Euros among those with expenditure	627	941	315	647	810	386	636	877	214	640	855	291
Mental health care (% yes)	6.7	418		4.4	356		2.0	89		4.6	863	
Expenditure in Euros among those with expenditure	4790	10,048	2346	3650	7149	1500	6291	18,143	1992	4474	10,206	2010
Physiotherapeutic care (% yes)	1.2	73		2.8	231		5.7	256		3.0	560	
Expenditure in Euros among those with expenditure	1111	1060	728	855	857	639	1152	1072	827	1024	995	732
Home care (% yes)	0.4	26		1.3	107		5.5	248		2.0	381	
Expenditure in Euros among those with expenditure	6762	13,552	879	5451	8900	1367	4736	6956	1437	5075	8120	1367
Pharmaceutical care (% yes)	60.2	3780		74.5	6082		89.5	4028		73.4	13,890	
Expenditure in Euros among those with expenditure	229	1296	55	459	2130	112	599	1444	263	437	1751	116
Maternity home care (% yes)	5.1	318		-	-		-	-		1.7	318	
Expenditure in Euros among those with expenditure	1323	952	1653	-	-	-	-	-	-	1323	952	1653
**Healthcare utilization**												
General practitioner (% yes)^a^	23.7	1488		28.4	2317		34.9	1570		28.4	5375	
Number of consultations among users	1.4	1.1	1.0	1.5	1.0	1.0	1.5	1.2	1.0	1.5	1.1	1.0
Medical specialist (% yes)^a^	13.3	837		17.3	1409		24.6	1108		17.7	3354	
Number of consultations among users	1.8	1.5	1.0	1.7	1.7	1.0	1.6	1.6	1.0	1.7	1.6	1.0
Dentist (% yes)^a^	17.3	1083		19.8	1619		17.3	780		18.4	3482	
Number of consultations among users	1.2	0.6	1.0	1.2	0.5	1.0	1.2	0.5	1.0	1.2	0.5	1.0
Overnight hospital admission (% yes)	5.3	332		6.8	556		11.9	534		7.5	1422	
Number of admissions among users	1.5	1.5	1.0	1.8	4.7	1.0	2.0	4.6	1.0	1.8	4.1	1.0
Daytime hospital admission (% yes)	7.2	453		9.0	731		11.1	500		8.9	1684	
Number of admissions among users	1.9	5.1	1.0	1.7	2.3	1.0	2.3	6.7	1.0	1.9	4.8	1.0
Mental health professional (% yes)	13.4	843		8.8	722		3.7	167		9.1	1732	
Number of consultations among users	13.5	18.4	8.0	10.1	13.3	6.0	7.7	11.7	4.0	11.5	16.0	6.0
Physiotherapist (% yes)	28.5	1789		32.3	2639		33.9	1525		31.4	5953	
Number of consultations among users	12.0	17.4	7.0	14.1	18.4	8.0	18.8	25.5	10.0	14.7	20.3	8.0
Home care (% yes)	4.0	254		3.1	255		9.7	438		5.0	947	

^a^Concerns healthcare utilization in the past 4 weeks. For all other types of care, the time window of healthcare utilization is 12 months

In line with the expenditure, the most frequently consulted healthcare professional was the GP with 28.4 % of the total study population consulting this professional in the past 4 weeks. In addition, respectively 17.7 and 18.4 % of the population consulted a medical specialist and dentist in the past 4 weeks. Furthermore, almost one third of the participants consulted a physiotherapist in the past 12 months. Home care was generally the least frequently used type of healthcare.

### Differences in healthcare expenditure and utilization by education and income

Tables 3 and 4 and Additional file 1a-c show the regression coefficients of the differences in probability and amount of healthcare expenditure/utilization by educational level and income. In general, for most types of healthcare, participants with lower educational levels and lower incomes had higher healthcare expenditure and used more healthcare compared to participants with the highest educational levels and highest incomes. For some of the healthcare types, these differences were found in the probability of expenditure/utilization, for some in the amount of expenditure/utilization among users, and for some in both.

**Table 3:** Regression coefficients of the differences in healthcare expenditure by educational level (low vs. high) and income (1st vs. 5th quintile) among participants aged 25–44 years, 45–64 years, and 65–79 years

		Participants aged 25–44 years						Participants aged 45–64 years						Participants aged 65–79 years						Total study population					
		**Educational level** ***Low vs. high***			**Income** ***1st vs. 5th quintile***			**Educational level** ***Low vs. high***			**Income** ***1st vs. 5th quintile***			**Educational level** ***Low vs. high***			**Income** ***1st vs. 5th quintile***			**Educational level** ***Low vs. high***			**Income** ***1st vs. 5th quintile***		
*Model*		*OR / RR + 95 % CI*			*OR / RR + 95 % CI*			*OR / RR + 95 % CI*			*OR / RR + 95 % CI*			*OR / RR + 95 % CI*			*OR / RR + 95 % CI*			*OR / RR + 95 % CI*			*OR / RR + 95 % CI*		
Total healthcare expenditure (amount among those with expenditure)	1	**1.82**	**1.46**	**2.27**	**1.96**	**1.72**	**2.23**	**2.47**	**2.14**	**2.86**	**2.51**	**2.25**	**2.80**	**1.60**	**1.39**	**1.84**	**1.56**	**1.36**	**1.77**	**2.10**	**1.91**	**2.30**	**2.06**	**1.92**	**2.21**
	2	1.17	0.95	1.44	1.10	0.97	1.24	1.08	0.95	1.24	**1.16**	**1.05**	**1.29**	1.09	0.96	1.24	1.07	0.95	1.21	**1.14**	**1.04**	**1.24**	**1.13**	**1.06**	**1.21**
General practitioner care (amount among those with expenditure)	1	**1.43**	**1.28**	**1.61**	**1.48**	**1.39**	**1.59**	**1.61**	**1.49**	**1.74**	**1.52**	**1.43**	**1.61**	**1.18**	**1.08**	**1.29**	**1.23**	**1.13**	**1.33**	**1.44**	**1.37**	**1.51**	**1.44**	**1.38**	**1.49**
	2	**1.19**	**1.06**	**1.33**	**1.22**	**1.14**	**1.31**	**1.16**	**1.07**	**1.25**	**1.14**	**1.08**	**1.21**	0.99	0.91	1.08	1.04	0.97	1.13	**1.12**	**1.06**	**1.17**	**1.14**	**1.10**	**1.19**
Hospital care (yes)	1	**1.72**	**1.30**	**2.28**	**1.52**	**1.29**	**1.79**	**2.00**	**1.62**	**2.48**	**1.28**	**1.10**	**1.49**	**1.77**	**1.28**	**2.44**	1.15	0.87	1.51	**1.87**	**1.62**	**2.17**	**1.35**	**1.22**	**1.50**
	2	1.27	0.95	1.71	1.12	0.95	1.33	1.16	0.93	1.46	**0.74**	**0.63**	**0.87**	NA	NA	NA	NA	NA	NA	**1.21**	**1.03**	**1.41**	**0.88**	**0.78**	**0.98**
Hospital care (amount among those with expenditure)	1	1.26	0.97	1.62	1.10	0.94	1.30	**1.86**	**1.58**	**2.19**	**1.94**	**1.70**	**2.22**	**1.49**	**1.26**	**1.75**	**1.34**	**1.14**	**1.56**	**1.71**	**1.53**	**1.90**	**1.51**	**1.38**	**1.65**
	2	0.97	0.75	1.26	**0.80**	**0.68**	**0.95**	1.00	0.85	1.18	1.10	0.96	1.26	1.06	0.90	1.25	1.01	0.87	1.17	1.06	0.96	1.17	0.98	0.90	1.06
Mental health care (yes)	1	**1.76**	**1.10**	**2.82**	**3.11**	**2.24**	**4.32**	**1.79**	**1.21**	**2.65**	**3.12**	**2.22**	**4.39**	0.75	0.37	1.54	1.37	0.70	2.69	**1.60**	**1.21**	**2.11**	**2.86**	**2.29**	**3.58**
	2	0.88	0.53	1.46	**1.58**	**1.11**	**2.25**	**0.64**	**0.41**	**0.99**	1.29	0.89	1.86	**0.44**	**0.20**	**0.93**	0.84	0.41	1.71	**0.69**	**0.51**	**0.94**	**1.35**	**1.06**	**1.71**
Mental health care (amount among those with expenditure)	1	1.32	0.81	2.17	1.35	0.96	1.89	**1.68**	**1.14**	**2.48**	NA	NA	NA	1.33	0.56	3.14	1.64	0.69	3.87	**1.41**	**1.06**	**1.88**	**1.83**	**1.45**	**2.31**
	2	1.14	0.72	1.79	1.18	0.86	1.64	1.29	0.86	1.91	NA	NA	NA	0.89	0.37	2.15	0.99	0.37	2.63	1.13	0.85	1.49	**1.49**	**1.19**	**1.88**

*CI* confidence interval; *NA* not applicable (due to no or limited number of cases); *OR* odds ratio; *RR* rate ratio. Model 1: adjusted for age, sex, interaction between age and sex, and year of participation in the health survey. Model 2: model 1 + adjusted for general health, physical limitations, and mental health. ORs apply to part 1 outcomes (probability of healthcare expenditure) calculated using logistic regression, RRs apply to part 2 outcomes (amount of healthcare expenditure among those who used healthcare resources) calculated using GLM with log link function and gamma distribution. Bold indicates *p* < 0.05

**Table 4:** Regression coefficients of the differences in healthcare utilization by educational level (low vs. high) and income (1st vs. 5th quintile) among participants aged 25–44 years, 45–64 years, and 65–79 years

		Participants aged 25–44 years						Participants aged 45–64 years						Participants aged 65–79 years						Total study population					
		**Educational level** ***Low vs. high***			**Income** ***1st vs. 5th quintile***			**Educational level** ***Low vs. high***			**Income** ***1st vs. 5th quintile***			**Educational level** ***Low vs. high***			**Income** ***1st vs. 5th quintile***			**Educational level** ***Low vs. high***			**Income** ***1st vs. 5th quintile***		
*Model*		*OR / RR + 95 % CI*			*OR / RR + 95 % CI*			*OR / RR + 95 % CI*			*OR / RR + 95 % CI*			*OR / RR + 95 % CI*			*OR / RR + 95 % CI*			*OR / RR + 95 % CI*			*OR / RR + 95 % CI*		
General practitioner (yes)	1	**1.96**	**1.46**	**2.62**	**1.79**	**1.49**	**2.16**	**1.61**	**1.32**	**1.95**	**1.63**	**1.40**	**1.90**	**1.71**	**1.39**	**2.12**	**1.36**	**1.12**	**1.66**	**1.73**	**1.53**	**1.96**	**1.60**	**1.45**	**1.77**
	2	1.32	0.97	1.79	1.21	0.99	1.47	0.89	0.72	1.10	0.93	0.79	1.10	**1.28**	**1.02**	**1.59**	0.97	0.79	1.19	1.11	0.97	1.27	1.02	0.92	1.14
Number of consultations among users	1	**1.24**	**1.03**	**1.50**	**1.32**	**1.16**	**1.52**	**1.25**	**1.10**	**1.42**	**1.19**	**1.07**	**1.32**	1.10	0.95	1.26	1.11	0.97	1.26	**1.18**	**1.09**	**1.28**	**1.20**	**1.12**	**1.29**
	2	1.05	0.87	1.28	1.14	0.99	1.31	1.08	0.94	1.23	1.02	0.92	1.14	1.00	0.87	1.15	0.98	0.85	1.12	1.03	0.95	1.12	1.04	0.96	1.11
Medical specialist (yes)	1	**1.68**	**1.18**	**2.40**	1.20	0.96	1.50	**1.73**	**1.39**	**2.16**	**1.69**	**1.41**	**2.03**	1.19	0.95	1.50	1.21	0.97	1.50	**1.52**	**1.32**	**1.75**	**1.39**	**1.24**	**1.57**
	2	0.94	0.64	1.39	**0.65**	**0.51**	**0.83**	0.79	0.62	1.01	0.78	0.63	0.95	0.79	0.62	1.01	**0.77**	**0.61**	**0.97**	**0.83**	**0.71**	**0.97**	**0.74**	**0.65**	**0.84**
Number of consultations among users	1	0.96	0.74	1.26	1.16	0.98	1.36	**1.24**	**1.06**	**1.45**	**1.24**	**1.08**	**1.42**	1.03	0.87	1.21	1.05	0.89	1.24	**1.12**	**1.01**	**1.24**	**1.16**	**1.06**	**1.27**
	2	0.81	0.62	1.06	0.91	0.77	1.08	1.00	0.85	1.18	0.99	0.86	1.15	0.98	0.83	1.15	0.98	0.83	1.15	0.98	0.88	1.08	0.98	0.89	1.07
Daytime hospital admission (yes)	1	**1.68**	**1.06**	**2.66**	**1.56**	**1.15**	**2.10**	**1.62**	**1.21**	**2.17**	**1.31**	**1.03**	**1.68**	1.15	0.84	1.58	**1.38**	**1.02**	**1.87**	**1.48**	**1.22**	**1.79**	**1.40**	**1.19**	**1.64**
	2	1.19	0.74	1.92	1.10	0.80	1.52	0.99	0.72	1.34	0.77	0.59	1.00	0.84	0.61	1.17	0.99	0.72	1.35	0.98	0.80	1.19	0.91	0.77	1.08
Number of admissions among users	1	0.81	0.49	1.32	1.07	0.80	1.43	0.91	0.71	1.17	0.90	0.73	1.12	1.11	0.80	1.55	1.24	0.92	1.68	1.00	0.83	1.20	1.07	0.92	1.24
	2	0.71	0.44	1.14	0.80	0.61	1.07	**0.73**	**0.56**	**0.94**	**0.72**	**0.57**	**0.90**	0.83	0.60	1.15	0.97	0.72	1.31	**0.76**	**0.63**	**0.91**	**0.83**	**0.71**	**0.97**
Mental health professional (yes)	1	1.29	0.88	1.89	**2.67**	**2.12**	**3.37**	1.33	0.99	1.79	**2.08**	**1.64**	**2.64**	0.78	0.44	1.39	1.08	0.66	1.77	1.22	0.99	1.52	**2.19**	**1.87**	**2.56**
	2	0.58	0.37	0.89	**1.37**	**1.07**	**1.77**	**0.55**	**0.40**	**0.77**	0.95	0.73	1.24	**0.48**	**0.26**	**0.88**	0.66	0.39	1.12	**0.55**	**0.43**	**0.69**	1.08	0.91	1.29
Number of consultations among users	1	1.29	0.91	1.84	1.13	0.91	1.41	1.21	0.93	1.58	1.06	0.86	1.32	0.64	0.35	1.16	1.13	0.69	1.87	1.17	0.96	1.43	1.11	0.96	1.28
	2	1.02	0.72	1.45	0.95	0.77	1.18	1.02	0.78	1.33	0.88	0.71	1.09	0.59	0.32	1.09	1.08	0.64	1.81	0.98	0.80	1.19	0.93	0.81	1.08

*CI* confidence interval; *NA* not applicable (due to no or limited number of cases); *OR* odds ratio; *RR* rate ratio. Model 1: adjusted for age, sex, interaction between age and sex, and year of participation in the health survey. Model 2: model 1 + adjusted for general health, physical limitations, and mental health. ORs apply to part 1 outcomes (probability of healthcare utilization) calculated using logistic regression, RRs apply to part 2 outcomes (amount of healthcare utilization among those who used healthcare resources) calculated using GLM with log link function and negative binomial. Bold indicates *p* < 0.05

### Healthcare expenditure

**Fig. 2:**
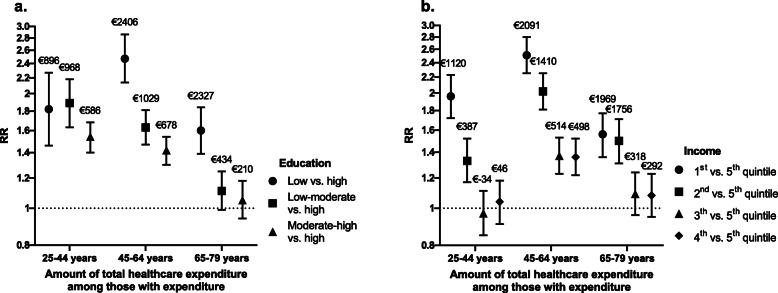
Regression coefficients of the differences in amount of total healthcare expenditure among those with expenditure by educational level (**a**) and income (**b**). Bars, 95 % confidence intervals; RR, rate ratio. Coefficients are adjusted for age, sex, interaction between age and sex, and year of participation in the health survey. The labels above the regression coefficients represent estimated marginal means of the difference in amount of total healthcare expenditure in Euros adjusted for covariates for each level of education and income compared to high education and the 5th income quintile, respectively

Among the participants with healthcare expenditure, the total amount of expenditure in Euros was, depending on age group, 60 % (rate ratio (RR) 1.60, 95 % CI 1.39–1.84) to 147 % (RR 2.47, 95 % CI 2.14–2.86) higher in those with the lowest educational levels compared to those with the highest educational levels (Fig. 2; Table 3). For those in the lowest compared to the highest income quintiles, this ranged from 56 % (RR 1.56, 95 % CI 1.36–1.77) to 151 % (RR 2.51, 95 % CI 2.25–2.80) (Fig. 2; Table 3). When translating the calculated rate ratios to actual Euros, the lowest educational levels had between €896 (for those aged 25–44 years) and €2406 (for those aged 45–64 years) higher healthcare expenditure than those with the highest educational levels. With respect to income, the lowest incomes had between €1120 (for those aged 25–44 years) and €2091 (for those aged 45–64 years) higher healthcare expenditure than those with the highest incomes. Participants with lower educational levels and lower incomes were found to have a higher probability of expenditure and/or amount of expenditure for GP care (amount), hospital care (probability and amount), dental care (probability), mental health care (probability and amount), home care (probability) and pharmaceutical care (probability and amount). For physiotherapeutic care and maternity home care, differences were less distinct or absent.

### Healthcare utilization

**Fig. 3:**
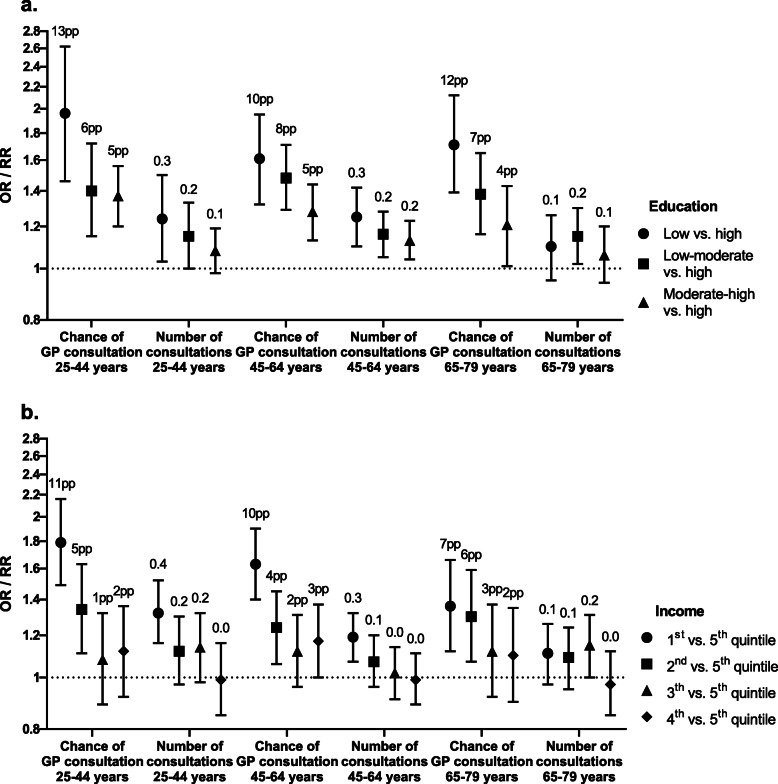
Regression coefficients of the differences in use of GP care by educational level (**a**) and income (**b**). Bars, 95 % confidence intervals; OR, odds ratio; pp, percentage point; RR, rate ratio. Coefficients are adjusted for age, sex, interaction between age and sex, and year of participation in the health survey. ORs apply to probability of GP consultation, RRs apply to number of consultations among users. The labels above the regression coefficients represent estimated marginal means of the difference in probability of GP consultation (in percentage points) and number of consultations adjusted for covariates for each level of education and income compared to high education and the 5th income quintile, respectively

Participants aged 25–44 years and 45–64 years in the lowest educational level or income quintile were more likely to consult a GP (odds ratio (OR) varying between 1.36 (95 % CI 1.12–1.66) and 1.96 (95 % CI 1.46–2.62)), and had more consultations (RR varying between 1.19 (95 % CI 1.07–1.32) and 1.32 (95 % CI 1.16–1.52)) than participants with the highest educational level and in the highest income quintile (Fig. 3; Table 4). This comes down to an approximately 10 % point higher probability of consulting a GP and 0.3 more consultations in 4 weeks among participants in the lowest educational level as well as among participants in the lowest income quintile compared to their counterparts in the highest educational and income groups. In addition, for medical specialist care, overnight and daytime hospital admission, mental health care, and home care, similar socioeconomic differences were found. The opposite result was found for physiotherapeutic care and dental care were participants in the lowest income quintile were generally less likely to consult a physiotherapist or dentist.

### The role of health in educational and income differences in healthcare expenditure and utilization

To examine whether health could explain the observed educational and income differences in healthcare expenditure and utilization, Tables 3 and 4 and Additional file 1a-c show the regression coefficients for these differences adjusted for general health, physical limitations, and mental health. While for most types of healthcare participants with lower educational levels and lower incomes had higher healthcare expenditure and used more healthcare, these differences generally disappeared or decreased after including the three indicators of health status in the analyses.

### Healthcare expenditure

**Fig. 4:**
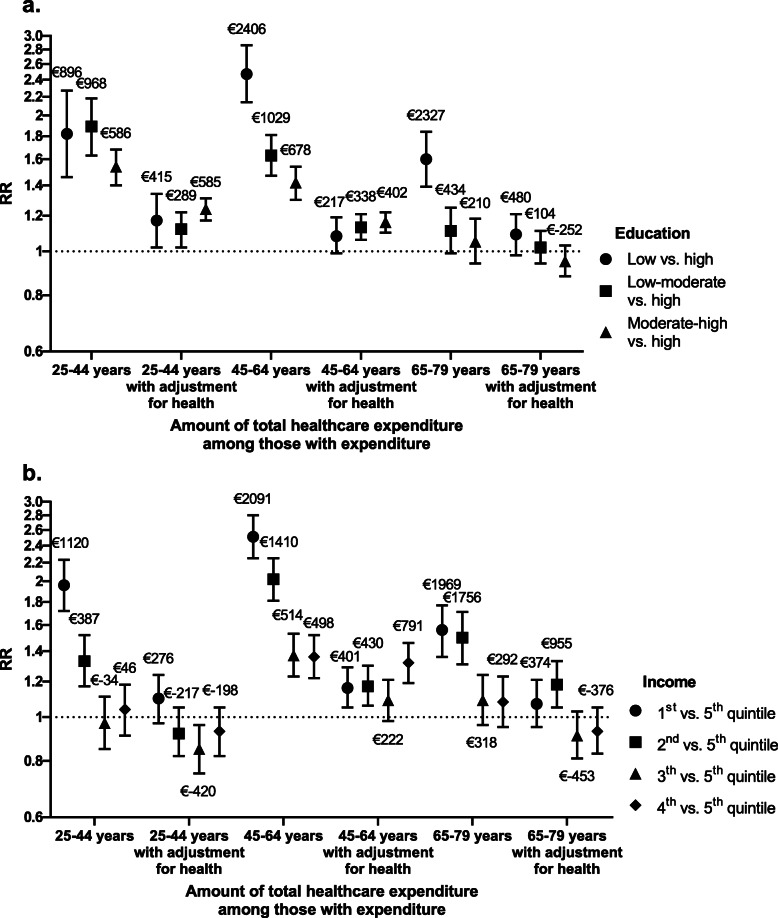
Regression coefficients of the differences in amount of total healthcare expenditure among those with expenditure by educational level (**a**) and income (**b**) with adjustment for health. Bars, 95 % confidence intervals; RR, rate ratio. Coefficients are adjusted for age, sex, interaction between age and sex, and year of participation in the health survey. Coefficients with adjustment for health are additionally adjusted for general health, physical limitations, and mental health. The labels above the regression coefficients represent estimated marginal means of the difference in amount of total healthcare expenditure in Euros adjusted for covariates for each level of education and income compared to high education and the 5th income quintile, respectively

In all age groups, the association between low socioeconomic status and higher total healthcare expenditure considerably declined or even disappeared after adjustment for health (Fig. 4; Table 3). This effect was most pronounced in participants aged 45–64 years in which the regression coefficients for the lowest educational level changed from 2.47 (95 % CI 2.14–2.86) to 1.08 (95 % CI 0.95–1.24), and for the lowest income quintile from 2.51 (95 % CI 2.25–2.80) to 1.16 (95 % CI 1.05–1.29). Socioeconomic differences diminished for all different types of healthcare after adjustment for health, but changes were most pronounced for GP care, hospital care, mental health care, home care, and pharmaceutical care.

### Healthcare utilization

**Fig. 5:**
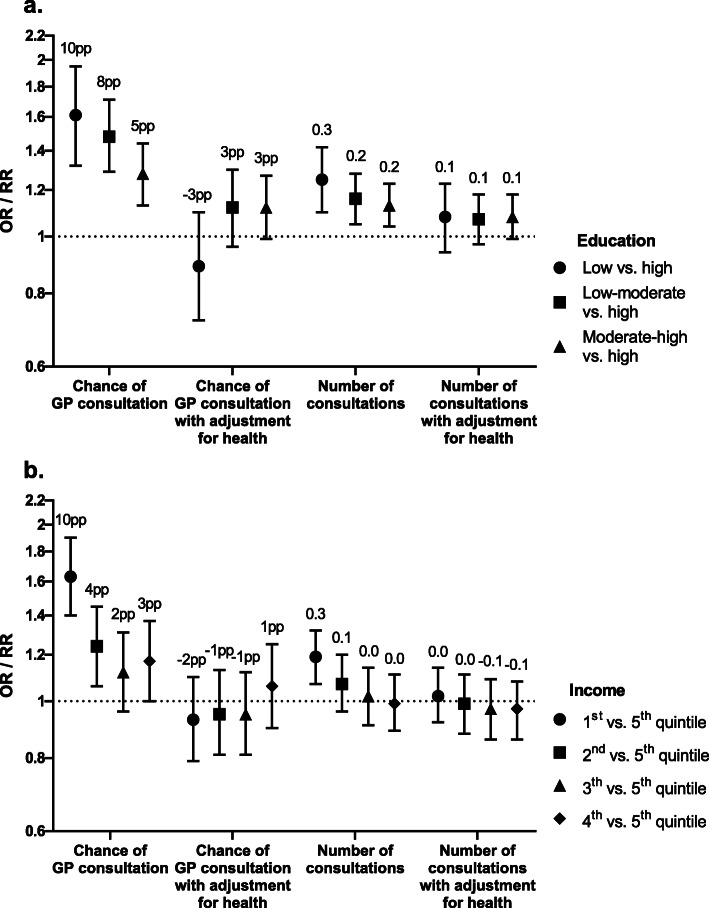
Regression coefficients of the differences in use of GP care by educational level (**a**) and income (**b**) level among participants aged 45–64 years with adjustment for health. Bars, 95 % confidence intervals; OR, odds ratio; pp, percentage point; RR, rate ratio. Coefficients are adjusted for age, sex, interaction between age and sex, and year of participation in the health survey. Coefficients with adjustment for health are additionally adjusted for general health, physical limitations, and mental health. ORs apply to probability of GP consultation, RRs apply to number of consultations among users. The labels above the regression coefficients represent estimated marginal means of the difference in probability of GP consultation (in percentage points) and number of consultations adjusted for covariates for each level of education and income compared to high education and the 5th income quintile, respectively

Figure 5 shows the regression coefficients for probability of consulting a GP and the number of consultations among users by educational level and income, with and without adjustment for health among participants aged 45–64 years. After adjustment for health, there were generally no differences in use of GP care by educational level and income. The odds ratio of consulting a GP in participants aged 45–64 years in the lowest compared to the highest educational level dropped from 1.61 (95 % CI 1.32–1.95) to 0.89 (95 % CI 0.72–1.10) after adjustment for health. Furthermore, for some types of healthcare that initially showed higher use among lower socioeconomic groups or no differences in use, lower use among lower socioeconomic groups appeared after including health. For example, the odds ratio of using home care in participants aged 65–79 years in the lowest educational level changed from 1.47 (95 % CI 1.05–2.04) without adjustment for health to 0.67 (95 % CI 0.46–0.97) with adjustment for health (Additional file 1c).

## Discussion

In this cross-sectional study among Dutch adults, people with a lower socioeconomic status, as indicated by a low educational and income level, had higher healthcare expenditure and used more healthcare than those with the highest educational and income level. Total healthcare expenditure was approximately 50 % (in those aged 65–79 years), 100 % (in those aged 25–44 years), and 150 % (in those aged 45–64 years) higher among people in the lowest educational as well as income levels. Increased use of healthcare resources among lower socioeconomic groups was generally found for both education and income, healthcare expenditure and utilization, different types of care, and probability as well as amount of use. However, the differences in healthcare expenditure and utilization by education and income level generally disappeared or strongly decreased after taking into account health status.

The findings of the current study are in line with previous studies that show higher healthcare expenditure and utilization among people with lower socioeconomic status. These studies have been conducted in different high-income countries with universal coverage systems, such as Canada [13, 14], England [10], and Sweden [15]. In the Netherlands, although there have been major reforms in the Dutch healthcare system over the years such as the introduction of the Health Insurance Act in 2006 and the Long-Term Care Act in 2015 [33], similar results have also been reported approximately 25 and 15 years ago [16, 34, 35]. In addition, there is evidence that sociodemographic differences in healthcare utilization in other European and Northern American countries have remained stable in the last decades [17]. In accordance with the present results, the previous Dutch studies also found socioeconomic inequalities in almost all types of healthcare. For visits to the GP and hospitalization, Kunst et al. (2007) observed one-and-a-half fold to two fold differences between the lowest and highest socioeconomic groups [16], which is comparable to the current findings. As has been observed earlier in a Canadian study [13], gradients were visible in the association of the different levels of education and income with healthcare expenditure and utilization. These gradients indicate that there is not only a difference between the lowest and highest educational and income levels, and that healthcare expenditure and utilization generally seem to increase as socioeconomic status decreases. The relative importance of educational differences versus income differences in health(care) is topic of discussion [36]. In the current study, differences by educational level were generally of a similar size as differences by income.

Our finding that the observed educational and income differences in healthcare expenditure and utilization largely diminished or disappeared after adjustment for health, has been widely reported [14–18]. Health status can be perceived as proxy for need for care and is a likely factor explaining the relation between socioeconomic status and healthcare expenditure/utilization. Because health status differs by socioeconomic status [3–5], it can be expected that people with a lower level of education and income have a poorer health and consequently utilize more healthcare resources than people on the other end of the socioeconomic gradient [5]. To illustrate, after adding variables for general health, physical limitations, and mental health, regression coefficients of the socioeconomic differences in total healthcare expenditure reduced by 74–91 % for the different age groups. These results indicate that to a large extent, health inequalities may explain the observed socioeconomic differences. In other words, socioeconomic inequality can be viewed as a determinant of health that subsequently contributes to differences in healthcare needs and healthcare expenditure. Our findings suggest that in the context of the Dutch healthcare system, those with higher healthcare needs generally also receive more care. This is probably due to the fact that the mandatory basic insurance package covers most necessary care.

In some instances, socioeconomic differences did not fully disappear after adjustment for health. This was mainly seen in associations that had rather large effect estimates to begin with and had therefore large variance to explain, such as probability of dental care and home care expenditure with ORs larger than 3. Explanations for this finding may be that additional health indicators are required to fully capture people’s need for healthcare services [37] or that other determinants are of importance for these types of care (e.g. health literacy). Future research is required to establish other mechanisms besides health status that could explain part of the increased healthcare expenditure among lower socioeconomic groups in such cases.

For some types of healthcare, differences showing lower use among lower socioeconomic groups appeared after adjusting for health covariates. For example, after adjustment for health, probabilities of receiving mental health care in participants aged 45–64 years and 65–79 years were lower for participants in the lowest compared to the highest educational levels. These findings are noteworthy, because they may indicate that with a similar health status, lower socioeconomic groups may have less access to this type of healthcare services. While results on mental health care are scarce, similar socioeconomic differences favoring higher socioeconomic groups have been observed across many countries for utilization of hospital care [20].

The association of lower socioeconomic status with increased healthcare expenditure and utilization was found across all three age groups (25–44 years, 45–64 years, 65–79 years). Although socioeconomic differences in for example total healthcare expenditure and GP care were rather consistent in the three age groups, the largest differences were generally found in participants aged 45–64 years. This may seem surprising, because need for healthcare increases with age, and thus the largest actual socioeconomic differences could be expected in the oldest age group. However, socioeconomic health inequalities have been found to expand until middle age, and to narrow post-retirement [5]. As inequalities in morbidity and mortality tend to be larger at middle compared to higher ages [4], this might implicate that the need for care of people with lower socioeconomic status follows the same pattern, subsequently resulting in larger relative socioeconomic differences in the middle age group.

For most types of healthcare, the observed socioeconomic differences in healthcare expenditure were reflected by comparable differences in healthcare utilization. Interestingly, for expenditure and utilization of dental care and physiotherapeutic care some opposite results were found in the age groups 45–64 years and 65–79 years. For example, participants aged 45–64 years in the lowest income quintile were more likely to have expenditure for dental care (OR = 5.00 (95 % CI 3.13-8.00), but less likely to visit a dentist (OR = 0.69 (95 % CI 0.58–0.82). However, this can be explained by the fact that dental care and physiotherapeutic care expenditure only represent expenditure covered by the basic insurance package, while visits to the dentist and physiotherapist for which supplementary insurance has to be purchased or that have to be paid out of pocket are not included in the expenditure outcomes. The fact that a large share of dental and physiotherapeutic care is not covered by the basic insurance package is also reflected by the percentage of participants with expenditure for dental care (3.5 %) and physiotherapeutic care (3.0 %), compared to the percentage of participants who reported to have visited a dentist (18.4 %) and physiotherapist (31.4 %). Subsequently, our findings may indicate that healthcare for which supplementary insurance has to be purchased is underrepresented in lower socioeconomic groups, while these groups are overrepresented in the same type of healthcare that is covered by the basic insurance package. However, future research that also includes healthcare expenditure data based on supplementary insurance is needed to confirm these hypotheses.

### Strengths and limitations

Strengths of the current study are that healthcare expenditure data at the individual level was derived from objective registration data sources and that total healthcare expenditure as well as expenditure for specific types of care were studied [25]. Although healthcare expenditure from supplementary insurance was not taken into account, the results of the current study are valuable, because the basic insurance package in the Netherlands is very comprehensive and covers all necessary healthcare. Furthermore, objective expenditure data was supplemented with self-reported information on healthcare utilization to gain further insight into socioeconomic inequalities in healthcare. Lastly, not one, but two important indicators of socioeconomic status, education and income, were studied [6], and multiple health indicators were included to explain the association between socioeconomic status and healthcare expenditure/utilization.

The current study was conducted in a large population-based sample. The fact that persons were randomly selected from the population to participate adds to the representativeness of the sample. However, selective non-response among certain social groups cannot be ruled out. In addition, differential reporting of healthcare utilization between older and younger participants is possible. Nonetheless, as separate analyses were conducted for the different age groups and as we have no reason to assume that differential reporting by age also differs by socioeconomic status, we believe the impact of this potential bias on our results to be limited. Furthermore, as data on socioeconomic status were derived from national registries, the expected bias due to incorrect reporting of educational or income level is probably minimal.

A limitation of the current study is its cross-sectional design. While we hypothesize that education and income influence health and subsequently healthcare expenditure and utilization, education and income are also known to be influenced by health status. Thus, besides a causal effect of socioeconomic status, there is also a possible selection effect in which people with worse health have fewer possibilities to obtain a higher educational and income level [4, 38]. Nonetheless, according to previous work, selection effects explain a smaller part of the association between education/income and health, and most evidence in public health literature supports the theory of causal effects [4, 38]. Still, for future work, it is recommended to also take into account possible selection effects. Another limitation of the cross-sectional design is that it only reflects socioeconomic differences at one moment in time. Yet, socioeconomic status changes over the life-course and these changes subsequently influence and are influenced by health status [36, 39]. Therefore, using a longitudinal design and lifetime perspective may complement our findings by giving an overview of the cumulative result of (changes in) socioeconomic status on healthcare expenditure/utilization over the life-course [5, 36].

In the current study, data on healthcare utilization were derived from DHIS 2016, 2017, and 2018, while healthcare expenditure data was based on nationwide claims data from 2017. Therefore, a note of caution is due when directly comparing the utilization and expenditure data. However, our aim was not to directly compare expenditure and utilization data at the individual level, but to gain insight into the extent healthcare expenditure and utilization differ by socioeconomic status, for which we believe our data were suitable.

The findings of this study also indicate that healthcare expenditure and utilization are skewed towards people of older age. However, the current study does not include the largest consumers of healthcare, i.e. persons aged 80 years and older. This group was excluded, because DHIS only includes participants living in private households. As a substantial part of the persons aged 80 years and older live in nursing home or other care facilities, the results for this age group would not have been representative for all elderly adults in the Netherlands. Nonetheless, especially in an ageing population, more research on inequalities in healthcare expenditure and utilization among elderly adults is needed. In such studies it may be relevant to explore other indicators for socioeconomic status and, besides health status, also include time to death as a potential mechanism explaining differences in both healthcare expenditure and utilization [40].

In the current study, we adjusted for health needs by using measures for general health, physical limitations, and mental health. However, it is uncertain whether these measures are sufficient to fully capture people’s need for healthcare services. Although perceived general health is widely accepted as a good predictor of healthcare utilization and mortality [17, 30], previous research indicates that higher socioeconomic groups may generally rate their health more negatively [37]. Therefore, the effect of adjusting for health on the association between education/income and healthcare expenditure/utilization may in reality be even larger than reported here. By also including measures for physical limitations and mental health, we have aimed to obtain a more complete overview of people’s health status. For most types of healthcare, the largest changes in effect estimates were observed after adjustment for general health and physical limitations, but additionally adjusting for mental health further reduced effect estimates for mental health care in particular.

## Conclusions

In 2007, Kunst et al. concluded that, in the Netherlands, “each euro spent on healthcare is used where it does the most good” [16]. Fifteen years later and after major reforms in the Dutch healthcare system, the current study still mainly supports this statement for individuals aged 25–79 years with different educational or income levels, as it shows that socioeconomic differences in healthcare expenditure and utilization largely disappear after taking into account health status. This implies that socioeconomic inequalities in health predominantly determine the increased use of healthcare resources among lower socioeconomic groups. For policy makers, health improvement efforts targeted at the low education and income groups may reduce health inequalities and healthcare spending.

## Supplementary Information

**Additional file 1:**

## Data Availability

This study was conducted using microdata sets of Statistics Netherlands (CBS) (https://www.cbs.nl/en-gb/our-services/customised-services-microdata/microdata-conducting-your-own-research). The data that support the findings of this study are available from Statistics Netherlands but restrictions apply to the availability of these data, which were used under license for the current study, and so are not publicly available. Data are however available from the authors upon reasonable request and with permission of Statistics Netherlands.

## Abbreviations

DHIS: Dutch Health Interview Survey
GLM: Generalized linear models
GP: General Practitioner
OECD: Organization for Economic Co-operation and Development
OR: Odds ratio
RR: Rate ratio
SD: Standard deviation
