# Supplementary material for: Socioeconomic differences in healthcare expenditure and utilization in The Netherlands
Source: BMC Health Serv Res. 2021 Jul 3;21:643. doi: 10.1186/s12913-021-06694-9 (PMC8254290; doi:10.1186/s12913-021-06694-9)
Supplement: Supplementary file 1 — Additional file 1: [file 12913_2021_6694_MOESM1_ESM.docx]

**Additional files**

Additional files of the manuscript entitled: “Socioeconomic differences in healthcare expenditure and utilization in The Netherlands”.

Authors: Bette Loef, Iris Meulman, Gerrie-Cor M. Herber, Geert Jan Kommer, Marc A. Koopmanschap, Anton E. Kunst, Johan J. Polder, Albert Wong, and Ellen Uiters.

Corresponding author: Bette Loef; E-mail: bette.loef@rivm.nl

**Additional file 1a.** Regression coefficients of the differences in healthcare expenditure and utilization by educational level and income among participants aged 25-44 years (n=6274)

|  |  | **Educational level** | | | | | | | | | **Income** | | | | | | | | | | | |
| --- | --- | --- | --- | --- | --- | --- | --- | --- | --- | --- | --- | --- | --- | --- | --- | --- | --- | --- | --- | --- | --- | --- |
|  |  | **Low vs. high** | | | **Low-moderate vs. high** | | | **Moderate-high vs. high** | | | **1^st^ vs. 5^th^ quintile** | | | **2^nd^ vs. 5^th^ quintile** | | | **3^th^ vs. 5^th^ quintile** | | | **4^th^ vs. 5^th^ quintile** | | |
| *Model* | | *OR / RR* | *95% CI* | | *OR / RR* | *95% CI* | | *OR / RR* | *95% CI* | | *OR / RR* | *95% CI* | | *OR / RR* | *95% CI* | | *OR / RR* | *95% CI* | | *OR / RR* | *95% CI* | |
| **Healthcare expenditure** | |  |  |  |  |  |  |  |  |  |  |  |  |  |  |  |  |  |  |  |  |  |
| Total healthcare expenditure (yes) | 1 | NA | NA | NA | NA | NA | NA | NA | NA | NA | NA | NA | NA | NA | NA | NA | NA | NA | NA | NA | NA | NA |
|  | 2 | NA | NA | NA | NA | NA | NA | NA | NA | NA | NA | NA | NA | NA | NA | NA | NA | NA | NA | NA | NA | NA |
| Amount among those with expenditure | 1 | **1.82** | **1.46** | **2.27** | **1.89** | **1.63** | **2.18** | **1.54** | **1.40** | **1.68** | **1.96** | **1.72** | **2.23** | **1.33** | **1.17** | **1.52** | 0.97 | 0.85 | 1.11 | 1.04 | 0.91 | 1.18 |
|  | 2 | 1.17 | 0.95 | 1.44 | 1.12 | 0.97 | 1.28 | **1.24** | **1.14** | **1.35** | 1.10 | 0.97 | 1.24 | 0.92 | 0.82 | 1.05 | **0.85** | **0.75** | **0.96** | 0.93 | 0.82 | 1.05 |
| General practitioner care (yes) | 1 | NA | NA | NA | NA | NA | NA | NA | NA | NA | NA | NA | NA | NA | NA | NA | NA | NA | NA | NA | NA | NA |
|  | 2 | NA | NA | NA | NA | NA | NA | NA | NA | NA | NA | NA | NA | NA | NA | NA | NA | NA | NA | NA | NA | NA |
| Amount among those with expenditure | 1 | **1.43** | **1.28** | **1.61** | **1.35** | **1.25** | **1.45** | **1.26** | **1.21** | **1.32** | **1.48** | **1.39** | **1.59** | **1.17** | **1.10** | **1.25** | **1.12** | **1.05** | **1.20** | 1.01 | 0.94 | 1.08 |
|  | 2 | **1.19** | **1.06** | **1.33** | **1.18** | **1.10** | **1.27** | **1.18** | **1.13** | **1.23** | **1.22** | **1.14** | **1.31** | 1.06 | 0.99 | 1.13 | 1.06 | 0.99 | 1.13 | 0.98 | 0.92 | 1.05 |
| Hospital care (yes) | 1 | **1.72** | **1.30** | **2.28** | **1.43** | **1.20** | **1.71** | **1.23** | **1.10** | **1.38** | **1.52** | **1.29** | **1.79** | 1.06 | 0.90 | 1.24 | 1.10 | 0.94 | 1.29 | 1.12 | 0.95 | 1.32 |
|  | 2 | 1.27 | 0.95 | 1.71 | 1.17 | 0.98 | 1.41 | 1.11 | 0.99 | 1.24 | 1.12 | 0.95 | 1.33 | 0.91 | 0.77 | 1.07 | 1.01 | 0.86 | 1.19 | 1.06 | 0.90 | 1.25 |
| Amount among those with expenditure | 1 | 1.26 | 0.97 | 1.62 | **1.22** | **1.02** | **1.45** | **1.33** | **1.18** | **1.49** | 1.10 | 0.94 | 1.30 | 1.00 | 0.84 | 1.18 | 0.85 | 0.72 | 1.00 | 0.95 | 0.81 | 1.13 |
|  | 2 | 0.97 | 0.75 | 1.26 | 0.97 | 0.81 | 1.16 | **1.19** | **1.06** | **1.33** | **0.80** | **0.68** | **0.95** | **0.84** | **0.72** | **1.00** | **0.79** | **0.67** | **0.93** | 0.90 | 0.77 | 1.06 |
| Dental care (yes) | 1 | NA | NA | NA | NA | NA | NA | NA | NA | NA | NA | NA | NA | NA | NA | NA | NA | NA | NA | NA | NA | NA |
|  | 2 | NA | NA | NA | NA | NA | NA | NA | NA | NA | NA | NA | NA | NA | NA | NA | NA | NA | NA | NA | NA | NA |
| Amount among those with expenditure | 1 | NA | NA | NA | NA | NA | NA | NA | NA | NA | NA | NA | NA | NA | NA | NA | NA | NA | NA | NA | NA | NA |
|  | 2 | NA | NA | NA | NA | NA | NA | NA | NA | NA | NA | NA | NA | NA | NA | NA | NA | NA | NA | NA | NA | NA |
| Mental health care (yes) | 1 | **1.76** | **1.10** | **2.82** | **1.60** | **1.16** | **2.20** | 1.22 | 0.98 | 1.52 | **3.11** | **2.24** | **4.32** | **1.66** | **1.16** | **2.38** | **1.45** | **1.00** | **2.09** | 1.17 | 0.80 | 1.71 |
|  | 2 | 0.88 | 0.53 | 1.46 | 0.98 | 0.70 | 1.39 | 0.95 | 0.75 | 1.20 | **1.58** | **1.11** | **2.25** | 1.15 | 0.79 | 1.67 | 1.22 | 0.83 | 1.77 | 1.09 | 0.74 | 1.61 |
| Amount among those with expenditure | 1 | 1.32 | 0.81 | 2.17 | **1.70** | **1.21** | **2.39** | 1.23 | 0.96 | 1.57 | 1.35 | 0.96 | 1.89 | 0.98 | 0.67 | 1.43 | 0.83 | 0.56 | 1.21 | **0.66** | **0.44** | **0.98** |
|  | 2 | 1.14 | 0.72 | 1.79 | **1.38** | **1.00** | **1.89** | 1.02 | 0.82 | 1.28 | 1.18 | 0.86 | 1.64 | 0.91 | 0.64 | 1.28 | 0.84 | 0.59 | 1.21 | **0.67** | **0.46** | **0.97** |
| Physiotherapeutic care (yes) | 1 | **2.94** | **1.18** | **7.33** | 1.85 | 0.87 | 3.93 | 1.57 | 0.92 | 2.67 | 1.81 | 0.83 | 3.95 | 1.31 | 0.57 | 2.99 | 1.89 | 0.87 | 4.08 | 1.30 | 0.57 | 2.99 |
|  | 2 | 1.45 | 0.54 | 3.89 | 1.28 | 0.59 | 2.78 | 1.33 | 0.78 | 2.30 | 0.91 | 0.39 | 2.10 | 0.96 | 0.41 | 2.25 | 1.59 | 0.73 | 3.47 | 1.15 | 0.50 | 2.64 |
| Amount among those with expenditure | 1 | 1.00 | 0.43 | 2.30 | 1.17 | 0.54 | 2.52 | 0.79 | 0.46 | 1.34 | 1.47 | 0.67 | 3.26 | 0.88 | 0.39 | 1.97 | 1.35 | 0.61 | 2.96 | 0.57 | 0.26 | 1.27 |
|  | 2 | 0.63 | 0.31 | 1.30 | 0.79 | 0.39 | 1.62 | 0.85 | 0.52 | 1.39 | 0.74 | 0.33 | 1.66 | 0.57 | 0.25 | 1.27 | 0.88 | 0.41 | 1.85 | 0.50 | 0.23 | 1.06 |
|  |  |  |  |  |  |  |  |  |  |  |  |  |  |  |  |  |  |  |  |  |  |  |
|  |  | **Educational level** | | | | | | | | | **Income** | | | | | | | | | | | |
|  |  | **Low vs. high** | | | **Low-moderate vs. high** | | | **Low vs. high** | | | **1^st^ vs. 5^th^ quintile** | | | **2^nd^ vs. 5^th^ quintile** | | | **3^th^ vs. 5^th^ quintile** | | | **4^th^ vs. 5^th^ quintile** | | |
| *Model* | | *OR / RR* | *95% CI* | | *OR / RR* | *95% CI* | | *OR / RR* | *95% CI* | | *OR / RR* | *95% CI* | | *OR / RR* | *95% CI* | | *OR / RR* | *95% CI* | | *OR / RR* | *95% CI* | |
| Home care (yes) | 1 | NA | NA | NA | NA | NA | NA | NA | NA | NA | NA | NA | NA | NA | NA | NA | NA | NA | NA | NA | NA | NA |
|  | 2 | NA | NA | NA | NA | NA | NA | NA | NA | NA | NA | NA | NA | NA | NA | NA | NA | NA | NA | NA | NA | NA |
| Amount among those with expenditure | 1 | NA | NA | NA | NA | NA | NA | NA | NA | NA | NA | NA | NA | NA | NA | NA | NA | NA | NA | NA | NA | NA |
|  | 2 | NA | NA | NA | NA | NA | NA | NA | NA | NA | NA | NA | NA | NA | NA | NA | NA | NA | NA | NA | NA | NA |
| Pharmaceutical care (yes) | 1 | **2.00** | **1.50** | **2.68** | **1.90** | **1.58** | **2.29** | **1.55** | **1.39** | **1.74** | **1.75** | **1.49** | **2.07** | **1.55** | **1.32** | **1.82** | **1.22** | **1.04** | **1.44** | **1.19** | **1.01** | **1.40** |
|  | 2 | **1.45** | **1.07** | **1.97** | **1.56** | **1.29** | **1.89** | **1.40** | **1.25** | **1.57** | **1.26** | **1.06** | **1.50** | **1.33** | **1.13** | **1.58** | 1.13 | 0.96 | 1.33 | 1.13 | 0.96 | 1.33 |
| Amount among those with expenditure | 1 | **1.38** | **1.09** | **1.74** | **1.12** | **0.96** | **1.30** | **1.42** | **1.28** | **1.58** | **2.77** | **2.40** | **3.19** | **1.45** | **1.25** | **1.67** | **1.33** | **1.15** | **1.53** | 1.13 | 0.98 | 1.31 |
|  | 2 | 1.11 | 0.90 | 1.37 | 0.91 | 0.79 | 1.04 | **1.36** | **1.24** | **1.50** | **1.56** | **1.36** | **1.79** | 1.03 | 0.90 | 1.18 | 0.99 | 0.86 | 1.13 | 1.01 | 0.88 | 1.16 |
| Maternity home care (yes) | 1 | 0.59 | 0.29 | 1.23 | **0.62** | **0.41** | **0.96** | **0.59** | **0.46** | **0.76** | 0.73 | 0.51 | 1.05 | **0.64** | **0.44** | **0.94** | 0.97 | 0.68 | 1.37 | 1.09 | 0.78 | 1.53 |
|  | 2 | 0.57 | 0.27 | 1.21 | **0.62** | **0.40** | **0.95** | **0.59** | **0.46** | **0.76** | 0.74 | 0.51 | 1.08 | **0.64** | **0.44** | **0.94** | 0.96 | 0.68 | 1.37 | 1.09 | 0.77 | 1.53 |
| Amount among those with expenditure | 1 | 0.79 | 0.34 | 1.82 | 0.67 | 0.41 | 1.09 | 0.99 | 0.74 | 1.33 | 0.88 | 0.57 | 1.35 | 1.05 | 0.66 | 1.65 | 1.07 | 0.71 | 1.63 | 0.99 | 0.67 | 1.46 |
|  | 2 | 0.80 | 0.34 | 1.88 | 0.71 | 0.42 | 1.19 | 1.00 | 0.75 | 1.34 | 0.94 | 0.58 | 1.53 | 1.05 | 0.66 | 1.66 | 1.08 | 0.71 | 1.66 | 0.99 | 0.66 | 1.47 |
| **Healthcare utilization** |  |  |  |  |  |  |  |  |  |  |  |  |  |  |  |  |  |  |  |  |  |  |
| General practitioner (yes) | 1 | **1.96** | **1.46** | **2.62** | **1.40** | **1.15** | **1.72** | **1.37** | **1.20** | **1.56** | **1.79** | **1.49** | **2.16** | **1.34** | **1.11** | **1.63** | 1.08 | 0.89 | 1.32 | 1.12 | 0.92 | 1.36 |
|  | 2 | 1.32 | 0.97 | 1.79 | 1.09 | 0.88 | 1.34 | **1.22** | **1.07** | **1.39** | 1.21 | 0.99 | 1.47 | 1.12 | 0.92 | 1.36 | 0.98 | 0.80 | 1.20 | 1.07 | 0.87 | 1.30 |
| Number of consul-tations among users | 1 | **1.24** | **1.03** | **1.50** | **1.15** | **1.00** | **1.33** | 1.08 | 0.98 | 1.19 | **1.32** | **1.16** | **1.52** | 1.12 | 0.97 | 1.30 | 1.14 | 0.98 | 1.32 | 0.99 | 0.85 | 1.16 |
|  | 2 | 1.05 | 0.87 | 1.28 | 1.05 | 0.91 | 1.21 | 1.03 | 0.94 | 1.14 | 1.14 | 0.99 | 1.31 | 1.04 | 0.90 | 1.21 | 1.07 | 0.92 | 1.25 | 0.97 | 0.83 | 1.13 |
| Medical specialist (yes) | 1 | **1.68** | **1.18** | **2.40** | 1.25 | 0.97 | 1.61 | **1.28** | **1.09** | **1.50** | 1.20 | 0.96 | 1.50 | 0.98 | 0.77 | 1.23 | 0.87 | 0.68 | 1.10 | 0.93 | 0.74 | 1.18 |
|  | 2 | 0.94 | 0.64 | 1.39 | 0.86 | 0.66 | 1.13 | 1.06 | 0.90 | 1.25 | **0.65** | **0.51** | **0.83** | **0.73** | **0.57** | **0.92** | **0.73** | **0.57** | **0.94** | 0.85 | 0.67 | 1.08 |
| Number of consul-tations among users | 1 | 0.96 | 0.74 | 1.26 | **1.22** | **1.02** | **1.46** | 1.10 | 0.98 | 1.24 | 1.16 | 0.98 | 1.36 | 0.99 | 0.83 | 1.18 | 1.14 | 0.95 | 1.36 | 0.97 | 0.81 | 1.16 |
|  | 2 | 0.81 | 0.62 | 1.06 | 1.08 | 0.90 | 1.29 | 1.03 | 0.91 | 1.16 | 0.91 | 0.77 | 1.08 | 0.88 | 0.74 | 1.05 | 1.04 | 0.87 | 1.24 | 0.95 | 0.80 | 1.13 |
| Dentist (yes) | 1 | 1.03 | 0.73 | 1.47 | 0.92 | 0.72 | 1.16 | 1.09 | 0.95 | 1.26 | 1.00 | 0.81 | 1.23 | 1.08 | 0.88 | 1.33 | 1.02 | 0.83 | 1.25 | 1.04 | 0.84 | 1.28 |
|  | 2 | 1.04 | 0.73 | 1.49 | 0.92 | 0.73 | 1.17 | 1.10 | 0.95 | 1.26 | 1.00 | 0.80 | 1.24 | 1.08 | 0.88 | 1.33 | 1.02 | 0.83 | 1.26 | 1.04 | 0.85 | 1.29 |
| Number of consul-tations among users | 1 | 1.09 | 0.81 | 1.45 | 1.09 | 0.90 | 1.33 | 1.05 | 0.93 | 1.18 | 1.05 | 0.87 | 1.25 | 1.06 | 0.89 | 1.27 | 1.03 | 0.86 | 1.23 | 1.03 | 0.86 | 1.23 |
|  | 2 | 1.02 | 0.76 | 1.38 | 1.08 | 0.88 | 1.32 | 1.05 | 0.93 | 1.18 | 1.02 | 0.85 | 1.23 | 1.05 | 0.88 | 1.26 | 1.02 | 0.86 | 1.23 | 1.03 | 0.86 | 1.23 |
| Overnight hospital admission (yes) | 1 | 1.65 | 0.96 | 2.85 | **1.87** | **1.32** | **2.65** | **1.34** | **1.05** | **1.72** | **1.51** | **1.08** | **2.12** | 1.11 | 0.77 | 1.58 | 0.96 | 0.66 | 1.38 | 0.89 | 0.61 | 1.30 |
|  | 2 | 1.03 | 0.58 | 1.82 | 1.43 | 1.00 | 2.05 | 1.14 | 0.89 | 1.47 | 0.98 | 0.68 | 1.40 | 0.90 | 0.63 | 1.30 | 0.85 | 0.59 | 1.24 | 0.83 | 0.57 | 1.22 |
|  |  | **Educational level** | | | | | | | | | **Income** | | | | | | | | | | | |
|  |  | **Low vs. high** | | | **Low-moderate vs. high** | | | **Low vs. high** | | | **1^st^ vs. 5^th^ quintile** | | | **2^nd^ vs. 5^th^ quintile** | | | **3^th^ vs. 5^th^ quintile** | | | **4^th^ vs. 5^th^ quintile** | | |
| *Model* | | *OR / RR* | *95% CI* | | *OR / RR* | *95% CI* | | *OR / RR* | *95% CI* | | *OR / RR* | *95% CI* | | *OR / RR* | *95% CI* | | *OR / RR* | *95% CI* | | *OR / RR* | *95% CI* | |
| Number of admissions among users | 1 | 0.92 | 0.56 | 1.50 | 1.30 | 0.98 | 1.74 | 1.06 | 0.86 | 1.32 | 1.21 | 0.92 | 1.58 | 0.82 | 0.60 | 1.13 | 0.95 | 0.70 | 1.30 | 0.78 | 0.56 | 1.10 |
|  | 2 | 0.97 | 0.59 | 1.57 | 1.21 | 0.91 | 1.62 | 0.98 | 0.79 | 1.22 | 1.05 | 0.79 | 1.39 | 0.76 | 0.55 | 1.04 | 0.94 | 0.69 | 1.28 | 0.77 | 0.55 | 1.08 |
| Daytime hospital admission (yes) | 1 | **1.68** | **1.06** | **2.66** | **1.56** | **1.14** | **2.13** | **1.29** | **1.05** | **1.60** | **1.56** | **1.15** | **2.10** | 1.28 | 0.94 | 1.75 | 1.09 | 0.79 | 1.51 | 1.03 | 0.74 | 1.42 |
|  | 2 | 1.19 | 0.74 | 1.92 | 1.25 | 0.90 | 1.72 | 1.13 | 0.91 | 1.41 | 1.10 | 0.80 | 1.52 | 1.09 | 0.80 | 1.50 | 1.00 | 0.72 | 1.38 | 0.97 | 0.70 | 1.34 |
| Number of admissions among users | 1 | 0.81 | 0.49 | 1.32 | 1.10 | 0.80 | 1.51 | 1.13 | 0.91 | 1.40 | 1.07 | 0.80 | 1.43 | 0.75 | 0.55 | 1.02 | 0.96 | 0.70 | 1.31 | **0.68** | **0.49** | **0.95** |
|  | 2 | 0.71 | 0.44 | 1.14 | 0.89 | 0.65 | 1.20 | 0.98 | 0.79 | 1.21 | 0.80 | 0.61 | 1.07 | **0.67** | **0.49** | **0.90** | 0.97 | 0.72 | 1.30 | 0.79 | 0.58 | 1.09 |
| Mental health professional (yes) | 1 | 1.29 | 0.88 | 1.89 | **1.41** | **1.11** | **1.80** | 1.16 | 0.99 | 1.36 | **2.67** | **2.12** | **3.37** | **1.34** | **1.04** | **1.72** | 1.23 | 0.96 | 1.59 | 1.09 | 0.84 | 1.42 |
|  | 2 | 0.58 | 0.37 | 0.89 | 0.86 | 0.66 | 1.13 | 0.92 | 0.77 | 1.09 | **1.37** | **1.07** | **1.77** | 0.91 | 0.70 | 1.19 | 1.03 | 0.79 | 1.35 | 1.01 | 0.77 | 1.33 |
| Number of consul-tations among users | 1 | 1.29 | 0.91 | 1.84 | **1.50** | **1.20** | **1.87** | **1.24** | **1.07** | **1.44** | 1.13 | 0.91 | 1.41 | 0.97 | 0.76 | 1.23 | 0.96 | 0.75 | 1.23 | 0.79 | 0.62 | 1.02 |
|  | 2 | 1.02 | 0.72 | 1.45 | 1.24 | 1.00 | 1.55 | 1.09 | 0.94 | 1.27 | 0.95 | 0.77 | 1.18 | 0.85 | 0.68 | 1.08 | 0.88 | 0.69 | 1.12 | **0.77** | **0.61** | **0.99** |
| Physiotherapist (yes) | 1 | 0.82 | 0.60 | 1.12 | 0.83 | 0.68 | 1.02 | 1.03 | 0.92 | 1.16 | **0.79** | **0.66** | **0.95** | 0.88 | 0.74 | 1.05 | 1.00 | 0.84 | 1.19 | 1.05 | 0.88 | 1.24 |
|  | 2 | **0.61** | **0.44** | **0.84** | **0.69** | **0.57** | **0.85** | 0.95 | 0.84 | 1.07 | **0.59** | **0.49** | **0.71** | **0.77** | **0.65** | **0.92** | 0.93 | 0.78 | 1.10 | 1.00 | 0.84 | 1.19 |
| Number of consul-tations among users | 1 | **1.32** | **1.03** | **1.70** | **1.21** | **1.03** | **1.42** | **1.22** | **1.11** | **1.34** | **1.43** | **1.24** | **1.65** | **1.19** | **1.03** | **1.37** | **1.25** | **1.09** | **1.43** | 1.07 | 0.93 | 1.22 |
|  | 2 | 1.02 | 0.81 | 1.29 | 0.92 | 0.79 | 1.07 | 1.07 | 0.98 | 1.17 | 0.89 | 0.77 | 1.02 | 0.97 | 0.85 | 1.11 | 1.04 | 0.91 | 1.18 | 0.93 | 0.82 | 1.05 |
| Home care (yes) | 1 | **3.36** | **2.00** | **5.63** | **1.73** | **1.13** | **2.64** | **1.47** | **1.10** | **1.95** | **2.27** | **1.55** | **3.33** | 0.67 | 0.41 | 1.09 | 1.34 | 0.88 | 2.04 | 0.92 | 0.58 | 1.45 |
|  | 2 | 1.61 | 0.89 | 2.92 | 1.10 | 0.69 | 1.74 | 1.20 | 0.89 | 1.62 | 1.27 | 0.83 | 1.93 | **0.50** | **0.30** | **0.83** | 1.19 | 0.77 | 1.84 | 0.84 | 0.53 | 1.33 |

CI, confidence interval; NA, not applicable (due to no or limited number of cases); OR, odds ratio; RR, rate ratio. Model 1: adjusted for age, sex, interaction between age and sex, and year of participation in the health survey. Model 2: model 1 + adjusted for general health, physical limitations, and mental health. ORs apply to part 1 outcomes (probability of healthcare expenditure/utilization) calculated using logistic regression, RRs apply to part 2 outcomes (amount of healthcare expenditure/utilization among those who used healthcare resources) calculated using GLM with log link function and gamma or negative binomial distribution. Bold indicates p<0.05.

**Additional file 1b.** Regression coefficients of the differences in healthcare expenditure and utilization by educational level and income among participants aged 45-64 years (n=8159)

|  |  | **Educational level** | | | | | | | | | **Income** | | | | | | | | | | | |
| --- | --- | --- | --- | --- | --- | --- | --- | --- | --- | --- | --- | --- | --- | --- | --- | --- | --- | --- | --- | --- | --- | --- |
|  |  | **Low vs. high** | | | **Low-moderate vs. high** | | | **Moderate-high vs. high** | | | **1^st^ vs. 5^th^ quintile** | | | **2^nd^ vs. 5^th^ quintile** | | | **3^th^ vs. 5^th^ quintile** | | | **4^th^ vs. 5^th^ quintile** | | |
| *Model* | | *OR / RR* | *95% CI* | | *OR / RR* | *95% CI* | | *OR / RR* | *95% CI* | | *OR / RR* | *95% CI* | | *OR / RR* | *95% CI* | | *OR / RR* | *95% CI* | | *OR / RR* | *95% CI* | |
| **Healthcare expenditure** | |  |  |  |  |  |  |  |  |  |  |  |  |  |  |  |  |  |  |  |  |  |
| Total healthcare expenditure (yes) | 1 | NA | NA | NA | NA | NA | NA | NA | NA | NA | NA | NA | NA | NA | NA | NA | NA | NA | NA | NA | NA | NA |
|  | 2 | NA | NA | NA | NA | NA | NA | NA | NA | NA | NA | NA | NA | NA | NA | NA | NA | NA | NA | NA | NA | NA |
| Amount among those with expenditure | 1 | **2.47** | **2.14** | **2.86** | **1.63** | **1.47** | **1.81** | **1.42** | **1.30** | **1.54** | **2.51** | **2.25** | **2.80** | **2.02** | **1.81** | **2.25** | **1.37** | **1.23** | **1.53** | **1.36** | **1.22** | **1.52** |
|  | 2 | 1.08 | 0.95 | 1.24 | **1.13** | **1.03** | **1.25** | **1.16** | **1.07** | **1.25** | **1.16** | **1.05** | **1.29** | **1.17** | **1.06** | **1.30** | 1.09 | 0.98 | 1.21 | **1.32** | **1.19** | **1.46** |
| General practitioner care (yes) | 1 | NA | NA | NA | NA | NA | NA | NA | NA | NA | NA | NA | NA | NA | NA | NA | NA | NA | NA | NA | NA | NA |
|  | 2 | NA | NA | NA | NA | NA | NA | NA | NA | NA | NA | NA | NA | NA | NA | NA | NA | NA | NA | NA | NA | NA |
| Amount among those with expenditure | 1 | **1.61** | **1.49** | **1.74** | **1.36** | **1.28** | **1.43** | **1.17** | **1.12** | **1.22** | **1.52** | **1.43** | **1.61** | **1.35** | **1.27** | **1.43** | **1.14** | **1.08** | **1.21** | **1.06** | **1.00** | **1.13** |
|  | 2 | **1.16** | **1.07** | **1.25** | **1.16** | **1.10** | **1.22** | **1.08** | **1.04** | **1.13** | **1.14** | **1.08** | **1.21** | **1.14** | **1.08** | **1.21** | 1.05 | 0.99 | 1.11 | 1.02 | 0.97 | 1.08 |
| Hospital care (yes) | 1 | **2.00** | **1.62** | **2.48** | **1.20** | **1.05** | **1.38** | **1.22** | **1.09** | **1.36** | **1.28** | **1.10** | **1.49** | 1.12 | 0.97 | 1.30 | 1.04 | 0.90 | 1.20 | **1.22** | **1.06** | **1.42** |
|  | 2 | 1.16 | 0.93 | 1.46 | 0.91 | 0.79 | 1.05 | 1.06 | 0.95 | 1.19 | **0.74** | **0.63** | **0.87** | 0.86 | 0.74 | 1.00 | 0.88 | 0.76 | 1.02 | 1.11 | 0.95 | 1.29 |
| Amount among those with expenditure | 1 | **1.86** | **1.58** | **2.19** | **1.45** | **1.28** | **1.64** | **1.22** | **1.11** | **1.36** | **1.94** | **1.70** | **2.22** | **1.90** | **1.66** | **2.17** | **1.28** | **1.12** | **1.46** | **1.33** | **1.16** | **1.52** |
|  | 2 | 1.00 | 0.85 | 1.18 | **1.14** | **1.00** | **1.28** | 1.07 | 0.97 | 1.19 | 1.10 | 0.96 | 1.26 | **1.27** | **1.11** | **1.45** | 1.12 | 0.99 | 1.28 | **1.36** | **1.20** | **1.55** |
| Dental care (yes) | 1 | **8.14** | **4.93** | **13.5** | **6.03** | **3.81** | **9.53** | **3.06** | **1.95** | **4.80** | **5.00** | **3.13** | **8.00** | **3.30** | **2.02** | **5.38** | **1.94** | **1.15** | **3.29** | 1.43 | 0.82 | 2.49 |
|  | 2 | **5.71** | **3.40** | **9.61** | **5.07** | **3.19** | **8.06** | **2.85** | **1.81** | **4.47** | **3.63** | **2.23** | **5.90** | **2.85** | **1.74** | **4.67** | **1.77** | **1.05** | **3.01** | 1.35 | 0.77 | 2.35 |
| Amount among those with expenditure | 1 | 1.37 | 0.78 | 2.41 | 1.19 | 0.70 | 2.03 | 1.31 | 0.78 | 2.21 | 1.15 | 0.68 | 1.94 | 0.94 | 0.54 | 1.64 | 0.84 | 0.47 | 1.51 | 0.82 | 0.44 | 1.51 |
|  | 2 | 1.18 | 0.67 | 2.07 | 0.99 | 0.58 | 1.70 | 1.09 | 0.65 | 1.83 | 1.18 | 0.69 | 2.02 | 1.04 | 0.59 | 1.85 | 0.96 | 0.52 | 1.75 | 0.97 | 0.52 | 1.82 |
| Mental health care (yes) | 1 | **1.79** | **1.21** | **2.65** | 1.19 | 0.86 | 1.64 | 1.17 | 0.90 | 1.51 | **3.12** | **2.22** | **4.39** | 1.39 | 0.95 | 2.04 | 0.99 | 0.66 | 1.50 | 1.33 | 0.91 | 1.96 |
|  | 2 | **0.64** | **0.41** | **0.99** | **0.69** | **0.49** | **0.98** | 0.90 | 0.69 | 1.18 | 1.29 | 0.89 | 1.86 | 0.90 | 0.60 | 1.35 | 0.76 | 0.50 | 1.16 | 1.16 | 0.78 | 1.72 |
| Amount among those with expenditure | 1 | **1.68** | **1.14** | **2.48** | **2.33** | **1.67** | **3.24** | **1.89** | **1.47** | **2.45** | NA | NA | NA | NA | NA | NA | NA | NA | NA | NA | NA | NA |
|  | 2 | 1.29 | 0.86 | 1.91 | **1.98** | **1.43** | **2.75** | **1.53** | **1.17** | **1.99** | NA | NA | NA | NA | NA | NA | NA | NA | NA | NA | NA | NA |
| Physiotherapeutic care (yes) | 1 | **1.77** | **1.07** | **2.91** | **1.89** | **1.29** | **2.75** | 1.29 | 0.91 | 1.82 | 1.52 | 0.99 | 2.33 | 1.30 | 0.83 | 2.01 | 1.33 | 0.86 | 2.07 | 1.13 | 0.72 | 1.78 |
|  | 2 | 0.71 | 0.42 | 1.22 | 1.22 | 0.82 | 1.80 | 1.02 | 0.71 | 1.45 | **0.62** | **0.39** | **0.98** | 0.79 | 0.50 | 1.24 | 0.98 | 0.62 | 1.54 | 0.94 | 0.59 | 1.50 |
| Amount among those with expenditure | 1 | 1.20 | 0.76 | 1.91 | 0.92 | 0.64 | 1.32 | 1.07 | 0.77 | 1.50 | 1.25 | 0.83 | 1.89 | 1.37 | 0.88 | 2.14 | 1.24 | 0.80 | 1.90 | 1.07 | 0.69 | 1.65 |
|  | 2 | 0.95 | 0.60 | 1.50 | 0.88 | 0.61 | 1.26 | 0.95 | 0.68 | 1.31 | 0.95 | 0.61 | 1.47 | 1.12 | 0.72 | 1.73 | 1.14 | 0.74 | 1.77 | 0.99 | 0.65 | 1.51 |
|  |  |  |  |  |  |  |  |  |  |  |  |  |  |  |  |  |  |  |  |  |  |  |
|  |  | **Educational level** | | | | | | | | | **Income** | | | | | | | | | | | |
|  |  | **Low vs. high** | | | **Low-moderate vs. high** | | | **Low vs. high** | | | **1^st^ vs. 5^th^ quintile** | | | **2^nd^ vs. 5^th^ quintile** | | | **3^th^ vs. 5^th^ quintile** | | | **4^th^ vs. 5^th^ quintile** | | |
| *Model* | | *OR / RR* | *95% CI* | | *OR / RR* | *95% CI* | | *OR / RR* | *95% CI* | | *OR / RR* | *95% CI* | | *OR / RR* | *95% CI* | | *OR / RR* | *95% CI* | | *OR / RR* | *95% CI* | |
| Home care (yes) | 1 | **6.76** | **3.67** | **12.5** | **2.61** | **1.42** | **4.79** | 1.41 | 0.78 | 2.53 | **11.6** | **4.61** | **29.2** | **5.79** | **2.22** | **15.1** | 2.55 | 0.89 | 7.26 | 1.91 | 0.64 | 5.71 |
|  | 2 | **1.98** | **1.02** | **3.85** | 1.28 | 0.67 | 2.43 | 1.03 | 0.56 | 1.88 | **3.61** | **1.38** | **9.42** | **3.15** | **1.18** | **8.41** | 1.70 | 0.58 | 4.92 | 1.41 | 0.46 | 4.30 |
| Amount among those with expenditure | 1 | 1.49 | 0.55 | 4.02 | 0.45 | 0.17 | 1.18 | 1.97 | 0.77 | 5.07 | 1.94 | 0.40 | 9.37 | 1.00 | 0.19 | 5.21 | 3.73 | 0.60 | 23.2 | 0.99 | 0.17 | 5.61 |
|  | 2 | 1.42 | 0.58 | 3.51 | 0.52 | 0.20 | 1.30 | 1.82 | 0.78 | 4.27 | 1.35 | 0.36 | 5.04 | 0.40 | 0.10 | 1.56 | 2.16 | 0.47 | 9.92 | 0.80 | 0.18 | 3.56 |
| Pharmaceutical care (yes) | 1 | **2.73** | **2.13** | **3.50** | **1.76** | **1.51** | **2.05** | **1.45** | **1.30** | **1.63** | **1.86** | **1.58** | **2.19** | **1.42** | **1.21** | **1.66** | **1.41** | **1.20** | **1.64** | **1.34** | **1.15** | **1.56** |
|  | 2 | **1.49** | **1.15** | **1.95** | **1.34** | **1.14** | **1.57** | **1.27** | **1.12** | **1.43** | 1.06 | 0.89 | 1.26 | 1.08 | 0.92 | 1.27 | **1.20** | **1.02** | **1.40** | **1.21** | **1.03** | **1.42** |
| Amount among those with expenditure | 1 | **1.86** | **1.61** | **2.14** | **1.45** | **1.31** | **1.62** | **1.24** | **1.13** | **1.35** | **1.58** | **1.41** | **1.78** | **1.53** | **1.35** | **1.72** | **1.26** | **1.12** | **1.42** | 0.91 | 0.80 | 1.02 |
|  | 2 | 0.92 | 0.80 | 1.05 | **1.11** | **1.00** | **1.22** | 1.05 | 0.97 | 1.14 | **0.86** | **0.77** | **0.96** | 0.91 | 0.81 | 1.01 | **0.88** | **0.79** | **0.98** | **0.84** | **0.76** | **0.94** |
| Maternity home care (yes) | 1 | NA | NA | NA | NA | NA | NA | NA | NA | NA | NA | NA | NA | NA | NA | NA | NA | NA | NA | NA | NA | NA |
|  | 2 | NA | NA | NA | NA | NA | NA | NA | NA | NA | NA | NA | NA | NA | NA | NA | NA | NA | NA | NA | NA | NA |
| Amount among those with expenditure | 1 | NA | NA | NA | NA | NA | NA | NA | NA | NA | NA | NA | NA | NA | NA | NA | NA | NA | NA | NA | NA | NA |
|  | 2 | NA | NA | NA | NA | NA | NA | NA | NA | NA | NA | NA | NA | NA | NA | NA | NA | NA | NA | NA | NA | NA |
| **Healthcare utilization** |  |  |  |  |  |  |  |  |  |  |  |  |  |  |  |  |  |  |  |  |  |  |
| General practitioner (yes) | 1 | **1.61** | **1.32** | **1.95** | **1.48** | **1.29** | **1.71** | **1.28** | **1.13** | **1.44** | **1.63** | **1.40** | **1.90** | **1.24** | **1.06** | **1.45** | 1.12 | 0.96 | 1.31 | 1.17 | 1.00 | 1.37 |
|  | 2 | 0.89 | 0.72 | 1.10 | 1.12 | 0.96 | 1.30 | 1.12 | 0.99 | 1.27 | 0.93 | 0.79 | 1.10 | 0.95 | 0.81 | 1.13 | 0.95 | 0.81 | 1.12 | 1.06 | 0.90 | 1.25 |
| Number of consul-tations among users | 1 | **1.25** | **1.10** | **1.42** | **1.16** | **1.05** | **1.28** | **1.13** | **1.04** | **1.23** | **1.19** | **1.07** | **1.32** | 1.07 | 0.96 | 1.20 | 1.02 | 0.91 | 1.14 | 0.99 | 0.89 | 1.11 |
|  | 2 | 1.08 | 0.94 | 1.23 | 1.07 | 0.97 | 1.18 | 1.08 | 0.99 | 1.18 | 1.02 | 0.92 | 1.14 | 0.99 | 0.88 | 1.11 | 0.97 | 0.86 | 1.09 | 0.97 | 0.86 | 1.08 |
| Medical specialist (yes) | 1 | **1.73** | **1.39** | **2.16** | **1.29** | **1.09** | **1.53** | **1.27** | **1.10** | **1.46** | **1.69** | **1.41** | **2.03** | **1.27** | **1.05** | **1.54** | 1.16 | 0.96 | 1.41 | **1.23** | **1.02** | **1.49** |
|  | 2 | 0.79 | 0.62 | 1.01 | 0.85 | 0.71 | 1.03 | 1.05 | 0.90 | 1.21 | 0.78 | 0.63 | 0.95 | 0.87 | 0.71 | 1.06 | 0.91 | 0.75 | 1.12 | 1.06 | 0.87 | 1.30 |
| Number of consul-tations among users | 1 | **1.24** | **1.06** | **1.45** | 1.01 | 0.89 | 1.16 | 1.06 | 0.95 | 1.18 | **1.24** | **1.08** | **1.42** | 1.11 | 0.95 | 1.28 | 1.02 | 0.87 | 1.18 | 1.03 | 0.89 | 1.20 |
|  | 2 | 1.00 | 0.85 | 1.18 | 0.92 | 0.80 | 1.05 | 1.00 | 0.89 | 1.11 | 0.99 | 0.86 | 1.15 | 1.00 | 0.86 | 1.16 | 0.95 | 0.82 | 1.11 | 1.00 | 0.86 | 1.16 |
| Dentist (yes) | 1 | 0.82 | 0.65 | 1.04 | 0.90 | 0.77 | 1.06 | 0.94 | 0.82 | 1.06 | **0.69** | **0.58** | **0.82** | **0.80** | **0.67** | **0.95** | 0.93 | 0.78 | 1.10 | 0.86 | 0.73 | 1.02 |
|  | 2 | 0.83 | 0.66 | 1.06 | 0.90 | 0.77 | 1.06 | 0.94 | 0.82 | 1.06 | **0.67** | **0.56** | **0.81** | **0.79** | **0.66** | **0.94** | 0.92 | 0.78 | 1.09 | 0.86 | 0.73 | 1.02 |
| Number of consul-tations among users | 1 | **1.20** | **1.00** | **1.44** | 1.08 | 0.95 | 1.24 | 1.04 | 0.94 | 1.16 | 1.15 | 1.00 | 1.33 | 1.06 | 0.92 | 1.22 | 1.01 | 0.88 | 1.16 | 1.01 | 0.88 | 1.16 |
|  | 2 | 1.12 | 0.93 | 1.35 | 1.04 | 0.91 | 1.19 | 1.03 | 0.93 | 1.15 | 1.08 | 0.93 | 1.25 | 1.03 | 0.90 | 1.19 | 1.00 | 0.87 | 1.15 | 1.01 | 0.87 | 1.16 |
| Overnight hospital admission (yes) | 1 | **2.91** | **2.16** | **3.91** | **1.49** | **1.14** | **1.94** | **1.40** | **1.12** | **1.76** | **2.20** | **1.63** | **2.96** | **1.81** | **1.33** | **2.46** | **1.75** | **1.28** | **2.38** | **1.64** | **1.20** | **2.23** |
|  | 2 | **1.44** | **1.04** | **1.98** | 1.01 | 0.77 | 1.33 | 1.17 | 0.93 | 1.47 | 1.04 | 0.75 | 1.42 | 1.26 | 0.92 | 1.73 | **1.40** | **1.02** | **1.92** | **1.41** | **1.03** | **1.94** |
|  |  | **Educational level** | | | | | | | | | **Income** | | | | | | | | | | | |
|  |  | **Low vs. high** | | | **Low-moderate vs. high** | | | **Low vs. high** | | | **1^st^ vs. 5^th^ quintile** | | | **2^nd^ vs. 5^th^ quintile** | | | **3^th^ vs. 5^th^ quintile** | | | **4^th^ vs. 5^th^ quintile** | | |
| *Model* | | *OR / RR* | *95% CI* | | *OR / RR* | *95% CI* | | *OR / RR* | *95% CI* | | *OR / RR* | *95% CI* | | *OR / RR* | *95% CI* | | *OR / RR* | *95% CI* | | *OR / RR* | *95% CI* | |
| Number of admissions among users | 1 | 1.19 | 0.90 | 1.58 | **0.74** | **0.57** | **0.97** | 0.96 | 0.77 | 1.20 | 1.28 | 0.95 | 1.73 | **1.53** | **1.13** | **2.08** | 0.93 | 0.67 | 1.28 | 0.92 | 0.67 | 1.27 |
|  | 2 | 0.94 | 0.71 | 1.25 | **0.74** | **0.57** | **0.97** | 0.96 | 0.77 | 1.20 | 0.98 | 0.72 | 1.34 | 1.21 | 0.90 | 1.65 | 0.90 | 0.66 | 1.23 | 0.89 | 0.65 | 1.23 |
| Daytime hospital admission (yes) | 1 | **1.62** | **1.21** | **2.17** | **1.41** | **1.12** | **1.76** | **1.29** | **1.07** | **1.56** | **1.31** | **1.03** | **1.68** | 1.25 | 0.97 | 1.60 | 1.25 | 0.98 | 1.60 | 1.13 | 0.88 | 1.45 |
|  | 2 | 0.99 | 0.72 | 1.34 | 1.09 | 0.86 | 1.37 | 1.13 | 0.93 | 1.37 | 0.77 | 0.59 | 1.00 | 0.96 | 0.75 | 1.24 | 1.06 | 0.83 | 1.37 | 1.01 | 0.78 | 1.30 |
| Number of admissions among users | 1 | 0.91 | 0.71 | 1.17 | **0.77** | **0.63** | **0.94** | **0.82** | **0.70** | **0.98** | 0.90 | 0.73 | 1.12 | 1.00 | 0.80 | 1.24 | 0.88 | 0.71 | 1.10 | **0.72** | **0.57** | **0.90** |
|  | 2 | **0.73** | **0.56** | **0.94** | **0.69** | **0.56** | **0.84** | **0.76** | **0.64** | **0.89** | **0.72** | **0.57** | **0.90** | 0.91 | 0.73 | 1.13 | 0.85 | 0.68 | 1.06 | **0.71** | **0.57** | **0.90** |
| Mental health professional (yes) | 1 | 1.33 | 0.99 | 1.79 | 0.97 | 0.77 | 1.22 | 1.02 | 0.86 | 1.23 | **2.08** | **1.64** | **2.64** | 1.16 | 0.90 | 1.51 | 0.92 | 0.70 | 1.21 | 1.18 | 0.91 | 1.52 |
|  | 2 | **0.55** | **0.40** | **0.77** | **0.59** | **0.46** | **0.76** | 0.83 | 0.68 | 1.01 | 0.95 | 0.73 | 1.24 | 0.80 | 0.61 | 1.06 | **0.73** | **0.55** | **0.97** | 1.05 | 0.80 | 1.38 |
| Number of consul-tations among users | 1 | 1.21 | 0.93 | 1.58 | **1.24** | **1.00** | **1.54** | 1.16 | 0.98 | 1.37 | 1.06 | 0.86 | 1.32 | 1.11 | 0.87 | 1.41 | 1.01 | 0.79 | 1.30 | 0.91 | 0.72 | 1.15 |
|  | 2 | 1.02 | 0.78 | 1.33 | 1.14 | 0.92 | 1.40 | 1.11 | 0.94 | 1.30 | 0.88 | 0.71 | 1.09 | 0.95 | 0.75 | 1.20 | 0.92 | 0.72 | 1.17 | 0.80 | 0.63 | 1.01 |
| Physiotherapist (yes) | 1 | 0.96 | 0.79 | 1.17 | 1.05 | 0.92 | 1.21 | **1.13** | **1.01** | **1.26** | 0.95 | 0.82 | 1.10 | 0.90 | 0.78 | 1.05 | 0.96 | 0.83 | 1.11 | 1.11 | 0.96 | 1.29 |
|  | 2 | **0.64** | **0.52** | **0.79** | **0.86** | **0.75** | **0.99** | 1.02 | 0.91 | 1.14 | **0.63** | **0.54** | **0.74** | **0.74** | **0.63** | **0.86** | **0.84** | **0.72** | **0.98** | 1.03 | 0.89 | 1.19 |
| Number of consul-tations among users | 1 | **1.37** | **1.18** | **1.58** | **1.31** | **1.18** | **1.45** | **1.20** | **1.11** | **1.31** | **1.32** | **1.18** | **1.48** | **1.15** | **1.03** | **1.29** | **1.13** | **1.01** | **1.26** | 1.03 | 0.92 | 1.15 |
|  | 2 | 1.01 | 0.88 | 1.16 | 1.07 | 0.97 | 1.18 | **1.09** | **1.01** | **1.18** | 0.90 | 0.81 | 1.00 | 0.91 | 0.82 | 1.02 | 0.99 | 0.89 | 1.09 | 0.94 | 0.85 | 1.04 |
| Home care (yes) | 1 | **3.00** | **1.95** | **4.63** | **1.90** | **1.30** | **2.78** | **1.51** | **1.07** | **2.12** | **5.50** | **3.52** | **8.58** | **1.80** | **1.08** | **2.99** | 1.43 | 0.84 | 2.43 | 1.37 | 0.80 | 2.33 |
|  | 2 | 0.77 | 0.47 | 1.26 | 0.91 | 0.60 | 1.38 | 1.13 | 0.79 | 1.62 | **1.86** | **1.15** | **3.00** | 0.98 | 0.57 | 1.66 | 0.95 | 0.55 | 1.65 | 1.07 | 0.62 | 1.85 |

CI, confidence interval; NA, not applicable (due to no or limited number of cases); OR, odds ratio; RR, rate ratio. Model 1: adjusted for age, sex, interaction between age and sex, and year of participation in the health survey. Model 2: model 1 + adjusted for general health, physical limitations, and mental health. ORs apply to part 1 outcomes (probability of healthcare expenditure/utilization) calculated using logistic regression, RRs apply to part 2 outcomes (amount of healthcare expenditure/utilization among those who used healthcare resources) calculated using GLM with log link function and gamma or negative binomial distribution. Bold indicates p<0.05.

**Additional file 1c.** Regression coefficients of the differences in healthcare expenditure and utilization by educational level and income among participants aged 65-79 years (n=4503)

|  |  | **Educational level** | | | | | | | | | **Income** | | | | | | | | | | | |
| --- | --- | --- | --- | --- | --- | --- | --- | --- | --- | --- | --- | --- | --- | --- | --- | --- | --- | --- | --- | --- | --- | --- |
|  |  | **Low vs. high** | | | **Low-moderate vs. high** | | | **Moderate-high vs. high** | | | **1^st^ vs. 5^th^ quintile** | | | **2^nd^ vs. 5^th^ quintile** | | | **3^th^ vs. 5^th^ quintile** | | | **4^th^ vs. 5^th^ quintile** | | |
| *Model* | | *OR / RR* | *95% CI* | | *OR / RR* | *95% CI* | | *OR / RR* | *95% CI* | | *OR / RR* | *95% CI* | | *OR / RR* | *95% CI* | | *OR / RR* | *95% CI* | | *OR / RR* | *95% CI* | |
| **Healthcare expenditure** | |  |  |  |  |  |  |  |  |  |  |  |  |  |  |  |  |  |  |  |  |  |
| Total healthcare expenditure (yes) | 1 | NA | NA | NA | NA | NA | NA | NA | NA | NA | NA | NA | NA | NA | NA | NA | NA | NA | NA | NA | NA | NA |
|  | 2 | NA | NA | NA | NA | NA | NA | NA | NA | NA | NA | NA | NA | NA | NA | NA | NA | NA | NA | NA | NA | NA |
| Amount among those with expenditure | 1 | **1.60** | **1.39** | **1.84** | 1.11 | 0.99 | 1.25 | 1.05 | 0.94 | 1.18 | **1.56** | **1.36** | **1.77** | **1.50** | **1.31** | **1.71** | 1.09 | 0.96 | 1.24 | 1.08 | 0.95 | 1.23 |
|  | 2 | 1.09 | 0.96 | 1.24 | 1.02 | 0.92 | 1.13 | 0.95 | 0.86 | 1.06 | 1.07 | 0.95 | 1.21 | **1.18** | **1.05** | **1.33** | 0.91 | 0.81 | 1.03 | 0.93 | 0.83 | 1.05 |
| General practitioner care (yes) | 1 | NA | NA | NA | NA | NA | NA | NA | NA | NA | NA | NA | NA | NA | NA | NA | NA | NA | NA | NA | NA | NA |
|  | 2 | NA | NA | NA | NA | NA | NA | NA | NA | NA | NA | NA | NA | NA | NA | NA | NA | NA | NA | NA | NA | NA |
| Amount among those with expenditure | 1 | **1.18** | **1.08** | **1.29** | **1.14** | **1.06** | **1.22** | 1.04 | 0.97 | 1.11 | **1.23** | **1.13** | **1.33** | **1.18** | **1.09** | **1.28** | **1.10** | **1.02** | **1.19** | 1.03 | 0.95 | 1.12 |
|  | 2 | 0.99 | 0.91 | 1.08 | 1.07 | 1.00 | 1.14 | 1.01 | 0.95 | 1.08 | 1.04 | 0.97 | 1.13 | 1.06 | 0.98 | 1.14 | 1.02 | 0.94 | 1.10 | 1.00 | 0.92 | 1.08 |
| Hospital care (yes) | 1 | **1.77** | **1.28** | **2.44** | **1.27** | **1.01** | **1.61** | 1.22 | 0.97 | 1.52 | 1.15 | 0.87 | 1.51 | 1.20 | 0.91 | 1.58 | 1.08 | 0.83 | 1.40 | 1.10 | 0.84 | 1.43 |
|  | 2 | NA | NA | NA | NA | NA | NA | NA | NA | NA | NA | NA | NA | NA | NA | NA | NA | NA | NA | NA | NA | NA |
| Amount among those with expenditure | 1 | **1.49** | **1.26** | **1.75** | 1.00 | 0.87 | 1.15 | 1.02 | 0.89 | 1.16 | **1.34** | **1.14** | **1.56** | **1.44** | **1.23** | **1.68** | 1.10 | 0.94 | 1.28 | 1.07 | 0.92 | 1.25 |
|  | 2 | 1.06 | 0.90 | 1.25 | 0.94 | 0.83 | 1.08 | 0.90 | 0.79 | 1.02 | 1.01 | 0.87 | 1.17 | 1.12 | 0.97 | 1.30 | 0.90 | 0.78 | 1.04 | 0.87 | 0.75 | 1.01 |
| Dental care (yes) | 1 | **3.20** | **2.21** | **4.63** | **2.14** | **1.52** | **3.00** | **1.43** | **1.01** | **2.03** | **1.69** | **1.16** | **2.46** | **2.05** | **1.42** | **2.95** | **1.79** | **1.24** | **2.60** | 1.25 | 0.84 | 1.85 |
|  | 2 | **3.06** | **2.10** | **4.46** | **2.06** | **1.46** | **2.90** | 1.41 | 0.99 | 2.00 | **1.59** | **1.09** | **2.33** | **1.96** | **1.36** | **2.83** | **1.73** | **1.20** | **2.51** | 1.23 | 0.83 | 1.82 |
| Amount among those with expenditure | 1 | 1.42 | 0.91 | 2.22 | 0.98 | 0.65 | 1.47 | 1.14 | 0.75 | 1.75 | 0.87 | 0.56 | 1.36 | 1.10 | 0.72 | 1.68 | 0.95 | 0.62 | 1.46 | 0.97 | 0.61 | 1.54 |
|  | 2 | 1.37 | 0.87 | 2.15 | 0.95 | 0.62 | 1.45 | 1.13 | 0.73 | 1.73 | 0.83 | 0.53 | 1.30 | 1.12 | 0.73 | 1.71 | 0.94 | 0.61 | 1.44 | 0.96 | 0.61 | 1.52 |
| Mental health care (yes) | 1 | 0.75 | 0.37 | 1.54 | 0.64 | 0.35 | 1.17 | 0.98 | 0.57 | 1.69 | 1.37 | 0.70 | 2.69 | 1.21 | 0.61 | 2.42 | 1.37 | 0.70 | 2.68 | 0.77 | 0.36 | 1.66 |
|  | 2 | **0.44** | **0.20** | **0.93** | **0.50** | **0.27** | **0.94** | 0.87 | 0.50 | 1.53 | 0.84 | 0.41 | 1.71 | 0.83 | 0.41 | 1.69 | 1.12 | 0.56 | 2.25 | 0.71 | 0.32 | 1.55 |
| Amount among those with expenditure | 1 | 1.33 | 0.56 | 3.14 | 1.90 | 0.96 | 3.76 | 1.18 | 0.63 | 2.22 | 1.64 | 0.69 | 3.87 | 1.20 | 0.51 | 2.85 | 0.71 | 0.29 | 1.71 | 1.11 | 0.42 | 2.96 |
|  | 2 | 0.89 | 0.37 | 2.15 | 1.62 | 0.80 | 3.27 | 1.34 | 0.69 | 2.61 | 0.99 | 0.37 | 2.63 | 0.92 | 0.39 | 2.21 | 0.61 | 0.24 | 1.54 | 1.31 | 0.48 | 3.58 |
| Physiotherapeutic care (yes) | 1 | 1.27 | 0.82 | 1.97 | 1.21 | 0.83 | 1.76 | 1.23 | 0.85 | 1.79 | **1.62** | **1.03** | **2.55** | **2.08** | **1.34** | **3.22** | 1.56 | 0.98 | 2.47 | **1.59** | **1.00** | **2.52** |
|  | 2 | 0.69 | 0.43 | 1.10 | 0.95 | 0.64 | 1.41 | 1.08 | 0.73 | 1.58 | 0.87 | 0.54 | 1.40 | 1.37 | 0.86 | 2.16 | 1.20 | 0.75 | 1.93 | 1.36 | 0.85 | 2.18 |
| Amount among those with expenditure | 1 | 0.87 | 0.58 | 1.30 | 0.97 | 0.68 | 1.37 | **0.69** | **0.49** | **0.97** | 0.81 | 0.53 | 1.24 | 1.08 | 0.72 | 1.62 | 0.84 | 0.55 | 1.29 | 0.88 | 0.57 | 1.35 |
|  | 2 | 0.83 | 0.56 | 1.22 | 0.95 | 0.68 | 1.33 | **0.68** | **0.49** | **0.95** | 0.75 | 0.50 | 1.13 | 1.04 | 0.70 | 1.53 | 0.85 | 0.56 | 1.28 | 0.84 | 0.56 | 1.28 |
|  |  |  |  |  |  |  |  |  |  |  |  |  |  |  |  |  |  |  |  |  |  |  |
|  |  | **Educational level** | | | | | | | | | **Income** | | | | | | | | | | | |
|  |  | **Low vs. high** | | | **Low-moderate vs. high** | | | **Low vs. high** | | | **1^st^ vs. 5^th^ quintile** | | | **2^nd^ vs. 5^th^ quintile** | | | **3^th^ vs. 5^th^ quintile** | | | **4^th^ vs. 5^th^ quintile** | | |
| *Model* | | *OR / RR* | *95% CI* | | *OR / RR* | *95% CI* | | *OR / RR* | *95% CI* | | *OR / RR* | *95% CI* | | *OR / RR* | *95% CI* | | *OR / RR* | *95% CI* | | *OR / RR* | *95% CI* | |
| Home care (yes) | 1 | **2.83** | **1.80** | **4.46** | **2.06** | **1.36** | **3.14** | 1.16 | 0.74 | 1.83 | **4.14** | **2.49** | **6.90** | **3.09** | **1.83** | **5.21** | 1.66 | 0.94 | 2.93 | 1.60 | 0.90 | 2.87 |
|  | 2 | 1.42 | 0.87 | 2.32 | **1.67** | **1.07** | **2.60** | 0.97 | 0.60 | 1.56 | **2.14** | **1.24** | **3.67** | **1.92** | **1.10** | **3.34** | 1.21 | 0.66 | 2.20 | 1.35 | 0.74 | 2.48 |
| Amount among those with expenditure | 1 | 0.79 | 0.42 | 1.48 | 0.68 | 0.37 | 1.22 | **0.47** | **0.25** | **0.89** | 1.07 | 0.55 | 2.10 | 0.86 | 0.42 | 1.74 | 0.89 | 0.40 | 2.02 | 0.81 | 0.35 | 1.85 |
|  | 2 | 0.60 | 0.32 | 1.12 | 0.69 | 0.39 | 1.24 | **0.32** | **0.17** | **0.59** | 1.09 | 0.55 | 2.16 | 0.95 | 0.47 | 1.91 | 1.15 | 0.50 | 2.65 | 0.78 | 0.35 | 1.75 |
| Pharmaceutical care (yes) | 1 | **2.39** | **1.64** | **3.46** | **1.49** | **1.15** | **1.92** | **1.41** | **1.11** | **1.80** | **1.49** | **1.10** | **2.02** | **1.96** | **1.43** | **2.70** | **1.43** | **1.07** | **1.90** | 1.13 | 0.86 | 1.49 |
|  | 2 | NA | NA | NA | NA | NA | NA | NA | NA | NA | NA | NA | NA | NA | NA | NA | NA | NA | NA | NA | NA | NA |
| Amount among those with expenditure | 1 | **1.19** | **1.05** | **1.35** | 0.99 | 0.89 | 1.10 | 0.97 | 0.88 | 1.08 | **1.46** | **1.29** | **1.64** | **1.38** | **1.22** | **1.55** | **1.13** | **1.01** | **1.27** | **1.21** | **1.07** | **1.36** |
|  | 2 | **0.86** | **0.77** | **0.97** | **0.89** | **0.81** | **0.98** | 0.95 | 0.86 | 1.04 | NA | NA | NA | NA | NA | NA | NA | NA | NA | NA | NA | NA |
| Maternity home care (yes) | 1 | NA | NA | NA | NA | NA | NA | NA | NA | NA | NA | NA | NA | NA | NA | NA | NA | NA | NA | NA | NA | NA |
|  | 2 | NA | NA | NA | NA | NA | NA | NA | NA | NA | NA | NA | NA | NA | NA | NA | NA | NA | NA | NA | NA | NA |
| Amount among those with expenditure | 1 | NA | NA | NA | NA | NA | NA | NA | NA | NA | NA | NA | NA | NA | NA | NA | NA | NA | NA | NA | NA | NA |
|  | 2 | NA | NA | NA | NA | NA | NA | NA | NA | NA | NA | NA | NA | NA | NA | NA | NA | NA | NA | NA | NA | NA |
| **Healthcare utilization** |  |  |  |  |  |  |  |  |  |  |  |  |  |  |  |  |  |  |  |  |  |  |
| General practitioner (yes) | 1 | **1.71** | **1.39** | **2.12** | **1.38** | **1.16** | **1.65** | **1.21** | **1.01** | **1.43** | **1.36** | **1.12** | **1.66** | **1.30** | **1.07** | **1.59** | 1.12 | 0.92 | 1.37 | 1.10 | 0.90 | 1.35 |
|  | 2 | **1.28** | **1.02** | **1.59** | **1.23** | **1.02** | **1.48** | 1.12 | 0.93 | 1.34 | 0.97 | 0.79 | 1.19 | 1.04 | 0.84 | 1.28 | 0.97 | 0.79 | 1.19 | 1.02 | 0.83 | 1.25 |
| Number of consul-tations among users | 1 | 1.10 | 0.95 | 1.26 | **1.15** | **1.02** | **1.30** | 1.06 | 0.94 | 1.20 | 1.11 | 0.97 | 1.26 | 1.09 | 0.95 | 1.24 | **1.15** | **1.00** | **1.31** | 0.97 | 0.85 | 1.12 |
|  | 2 | 1.00 | 0.87 | 1.15 | 1.13 | 1.00 | 1.27 | 1.06 | 0.93 | 1.19 | 0.98 | 0.85 | 1.12 | 1.01 | 0.88 | 1.16 | 1.09 | 0.96 | 1.25 | 0.94 | 0.82 | 1.08 |
| Medical specialist (yes) | 1 | 1.19 | 0.95 | 1.50 | 0.98 | 0.81 | 1.19 | 0.96 | 0.79 | 1.16 | 1.21 | 0.97 | 1.50 | 1.15 | 0.92 | 1.43 | 1.00 | 0.80 | 1.25 | 1.08 | 0.87 | 1.34 |
|  | 2 | 0.79 | 0.62 | 1.01 | 0.82 | 0.67 | 1.01 | 0.86 | 0.70 | 1.04 | **0.77** | **0.61** | **0.97** | 0.86 | 0.68 | 1.08 | 0.82 | 0.65 | 1.04 | 0.97 | 0.77 | 1.22 |
| Number of consul-tations among users | 1 | 1.03 | 0.87 | 1.21 | 1.00 | 0.87 | 1.16 | 1.04 | 0.91 | 1.20 | 1.05 | 0.89 | 1.24 | 1.13 | 0.96 | 1.33 | 1.05 | 0.89 | 1.24 | 1.15 | 0.98 | 1.35 |
|  | 2 | 0.98 | 0.83 | 1.15 | 0.99 | 0.86 | 1.13 | 1.02 | 0.89 | 1.17 | 0.98 | 0.83 | 1.15 | 1.07 | 0.91 | 1.25 | 1.02 | 0.86 | 1.20 | 1.14 | 0.97 | 1.34 |
| Dentist (yes) | 1 | **0.61** | **0.46** | **0.80** | **0.79** | **0.64** | **0.98** | **0.74** | **0.60** | **0.91** | **0.63** | **0.49** | **0.81** | **0.70** | **0.55** | **0.89** | 0.79 | 0.63 | 1.01 | 0.91 | 0.72 | 1.15 |
|  | 2 | **0.62** | **0.47** | **0.83** | **0.81** | **0.65** | **1.00** | **0.74** | **0.60** | **0.91** | **0.64** | **0.49** | **0.82** | **0.71** | **0.56** | **0.91** | 0.81 | 0.64 | 1.03 | 0.93 | 0.73 | 1.17 |
| Number of consul-tations among users | 1 | 1.17 | 0.93 | 1.47 | 0.99 | 0.83 | 1.19 | 1.06 | 0.90 | 1.26 | 1.03 | 0.83 | 1.27 | 1.04 | 0.85 | 1.28 | 1.06 | 0.87 | 1.29 | 1.02 | 0.84 | 1.23 |
|  | 2 | 1.14 | 0.91 | 1.44 | 0.99 | 0.82 | 1.18 | 1.07 | 0.90 | 1.26 | 0.98 | 0.79 | 1.23 | 1.03 | 0.83 | 1.27 | 1.04 | 0.86 | 1.27 | 1.01 | 0.83 | 1.22 |
| Overnight hospital admission (yes) | 1 | **1.73** | **1.27** | **2.35** | **1.34** | **1.02** | **1.75** | 1.28 | 0.98 | 1.66 | **2.18** | **1.62** | **2.94** | **1.79** | **1.32** | **2.43** | 1.31 | 0.96 | 1.80 | 1.11 | 0.80 | 1.54 |
|  | 2 | 1.18 | 0.85 | 1.63 | 1.15 | 0.87 | 1.52 | 1.17 | 0.89 | 1.54 | **1.47** | **1.08** | **2.01** | **1.38** | **1.01** | **1.90** | 1.11 | 0.80 | 1.54 | 1.01 | 0.72 | 1.41 |
|  |  | **Educational level** | | | | | | | | | **Income** | | | | | | | | | | | |
|  |  | **Low vs. high** | | | **Low-moderate vs. high** | | | **Low vs. high** | | | **1^st^ vs. 5^th^ quintile** | | | **2^nd^ vs. 5^th^ quintile** | | | **3^th^ vs. 5^th^ quintile** | | | **4^th^ vs. 5^th^ quintile** | | |
| *Model* | | *OR / RR* | *95% CI* | | *OR / RR* | *95% CI* | | *OR / RR* | *95% CI* | | *OR / RR* | *95% CI* | | *OR / RR* | *95% CI* | | *OR / RR* | *95% CI* | | *OR / RR* | *95% CI* | |
| Number of admissions among users | 1 | **0.61** | **0.45** | **0.81** | **0.71** | **0.56** | **0.92** | **0.76** | **0.60** | **0.98** | **1.37** | **1.02** | **1.84** | 1.30 | 0.96 | 1.76 | 1.15 | 0.84 | 1.59 | **1.86** | **1.35** | **2.55** |
|  | 2 | **0.64** | **0.48** | **0.86** | 0.81 | 0.63 | 1.04 | 0.82 | 0.64 | 1.05 | 1.32 | 0.98 | 1.77 | 1.26 | 0.93 | 1.71 | 1.18 | 0.86 | 1.62 | **1.75** | **1.28** | **2.39** |
| Daytime hospital admission (yes) | 1 | 1.15 | 0.84 | 1.58 | 1.03 | 0.79 | 1.35 | 1.03 | 0.80 | 1.34 | **1.38** | **1.02** | **1.87** | 1.15 | 0.85 | 1.57 | 1.30 | 0.96 | 1.76 | 0.98 | 0.71 | 1.34 |
|  | 2 | 0.84 | 0.61 | 1.17 | 0.90 | 0.69 | 1.19 | 0.95 | 0.73 | 1.24 | 0.99 | 0.72 | 1.35 | 0.92 | 0.67 | 1.26 | 1.14 | 0.84 | 1.54 | 0.91 | 0.66 | 1.25 |
| Number of admissions among users | 1 | 1.11 | 0.80 | 1.55 | 0.87 | 0.65 | 1.17 | 1.10 | 0.84 | 1.45 | 1.24 | 0.92 | 1.68 | **0.63** | **0.45** | **0.87** | 0.93 | 0.68 | 1.27 | **0.70** | **0.50** | **0.98** |
|  | 2 | 0.83 | 0.60 | 1.15 | 0.84 | 0.64 | 1.10 | 0.98 | 0.75 | 1.27 | 0.97 | 0.72 | 1.31 | **0.66** | **0.48** | **0.92** | 0.94 | 0.70 | 1.28 | 0.82 | 0.59 | 1.14 |
| Mental health professional (yes) | 1 | 0.78 | 0.44 | 1.39 | 1.04 | 0.67 | 1.61 | 1.11 | 0.73 | 1.71 | 1.08 | 0.66 | 1.77 | 1.26 | 0.78 | 2.02 | 0.87 | 0.52 | 1.44 | 0.78 | 0.47 | 1.31 |
|  | 2 | **0.48** | **0.26** | **0.88** | 0.85 | 0.54 | 1.34 | 1.00 | 0.64 | 1.56 | 0.66 | 0.39 | 1.12 | 0.88 | 0.53 | 1.44 | 0.69 | 0.40 | 1.17 | 0.72 | 0.42 | 1.23 |
| Number of consul-tations among users | 1 | 0.64 | 0.35 | 1.16 | 1.10 | 0.70 | 1.74 | 1.03 | 0.68 | 1.58 | 1.13 | 0.69 | 1.87 | 1.12 | 0.69 | 1.83 | 1.18 | 0.69 | 2.02 | 0.81 | 0.47 | 1.38 |
|  | 2 | 0.59 | 0.32 | 1.09 | 0.99 | 0.63 | 1.58 | 0.96 | 0.61 | 1.49 | 1.08 | 0.64 | 1.81 | 1.12 | 0.68 | 1.83 | 1.15 | 0.67 | 2.00 | 0.88 | 0.50 | 1.56 |
| Physiotherapist (yes) | 1 | 0.87 | 0.70 | 1.08 | 0.99 | 0.83 | 1.18 | 1.10 | 0.92 | 1.30 | **0.75** | **0.61** | **0.91** | 0.91 | 0.75 | 1.11 | 0.92 | 0.75 | 1.11 | 0.90 | 0.74 | 1.09 |
|  | 2 | **0.63** | **0.50** | **0.79** | 0.87 | 0.73 | 1.04 | 1.02 | 0.86 | 1.22 | **0.53** | **0.43** | **0.65** | **0.73** | **0.59** | **0.89** | **0.79** | **0.64** | **0.97** | 0.82 | 0.67 | 1.00 |
| Number of consul-tations among users | 1 | 1.04 | 0.87 | 1.24 | 1.00 | 0.86 | 1.15 | 0.96 | 0.84 | 1.11 | 1.13 | 0.96 | 1.33 | **1.35** | **1.15** | **1.57** | 0.97 | 0.83 | 1.14 | 0.97 | 0.83 | 1.14 |
|  | 2 | 0.85 | 0.72 | 1.01 | 1.01 | 0.88 | 1.15 | 0.98 | 0.86 | 1.11 | 0.91 | 0.78 | 1.06 | 1.07 | 0.92 | 1.24 | 0.89 | 0.77 | 1.03 | **0.84** | **0.72** | **0.97** |
| Home care (yes) | 1 | **1.47** | **1.05** | **2.04** | 1.05 | 0.78 | 1.41 | 0.91 | 0.67 | 1.24 | **1.76** | **1.27** | **2.43** | 1.24 | 0.88 | 1.74 | 0.95 | 0.67 | 1.37 | 1.01 | 0.71 | 1.44 |
|  | 2 | **0.67** | **0.46** | **0.97** | 0.76 | 0.55 | 1.05 | 0.73 | 0.52 | 1.02 | 0.81 | 0.57 | 1.17 | **0.65** | **0.45** | **0.95** | **0.65** | **0.44** | **0.96** | 0.79 | 0.53 | 1.16 |

CI, confidence interval; NA, not applicable (due to no or limited number of cases); OR, odds ratio; RR, rate ratio. Model 1: adjusted for age, sex, interaction between age and sex, and year of participation in the health survey. Model 2: model 1 + adjusted for general health, physical limitations, and mental health. ORs apply to part 1 outcomes (probability of healthcare expenditure/utilization) calculated using logistic regression, RRs apply to part 2 outcomes (amount of healthcare expenditure/utilization among those who used healthcare resources) calculated using GLM with log link function and gamma or negative binomial distribution. Bold indicates p<0.05.
